# Correction: *Cuscuta* seeds: Diversity and evolution, value for systematics/identification and exploration of allometric relationships

**DOI:** 10.1371/journal.pone.0235738

**Published:** 2020-07-01

**Authors:** The PLOS ONE Staff

A second attachment from the Author Response is omitted from the Peer Review History. It can be viewed as a Supporting Information file below. The publisher apologizes for the error.

## Supporting information

S1 File Response to Reviewers 1 (Rebuttal Letter). (DOCX)
