# Supplementary figures and images for: Cuscuta seeds: Diversity and evolution, value for systematics/identification and exploration of allometric relationships
Source: PLoS One. 2020 Jun 12;15(6):e0234627. doi: 10.1371/journal.pone.0234627 (PMC7292398; doi:10.1371/journal.pone.0234627)

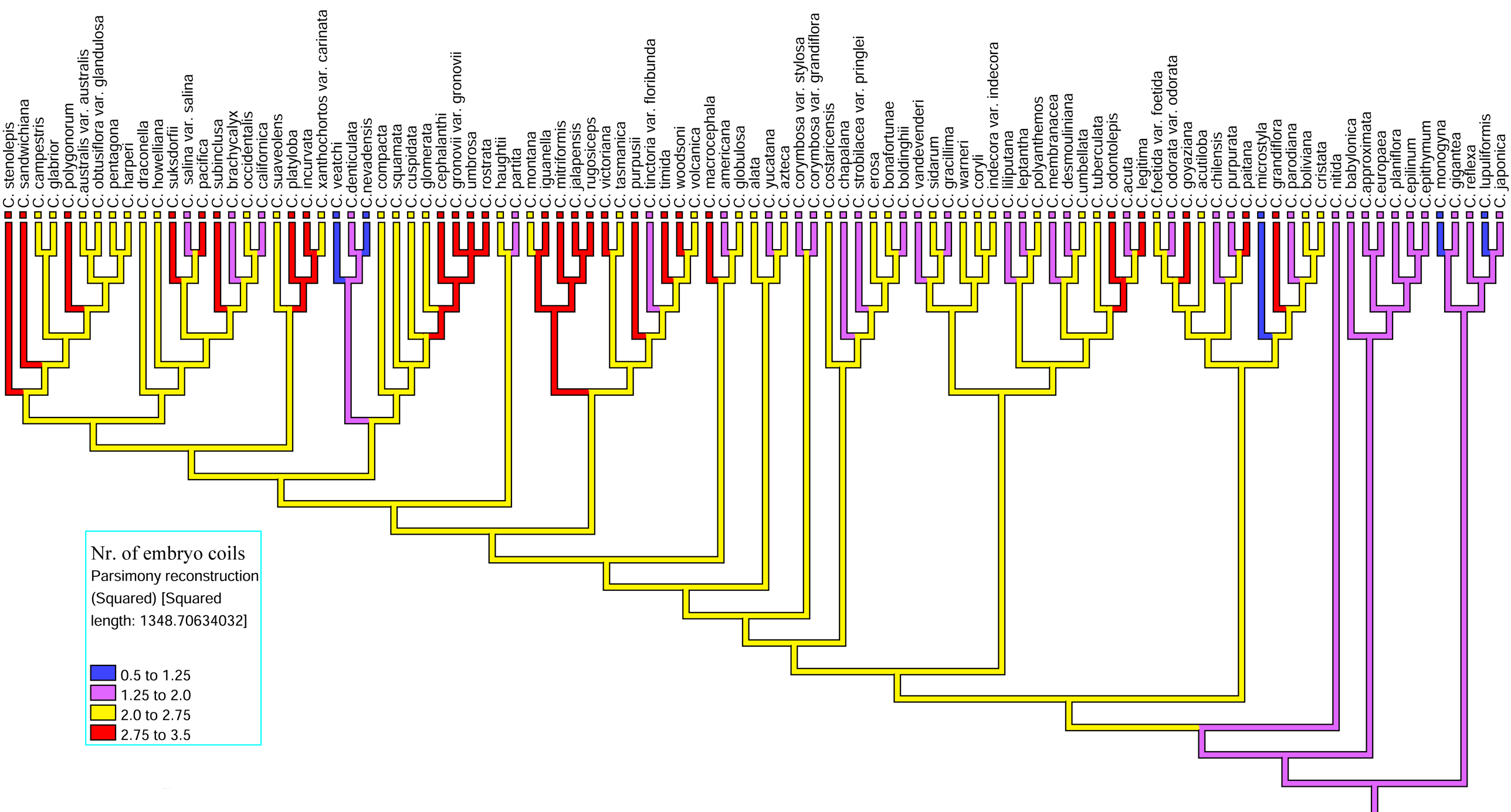

Supplement: S1 Fig — Embryos with more than 2.75 coils evolved multiple times in subg. Grammica. (TIF) [file pone.0234627.s001.tif]
