# Supplementary material for: Cuscuta seeds: Diversity and evolution, value for systematics/identification and exploration of allometric relationships
Source: PLoS One. 2020 Jun 12;15(6):e0234627. doi: 10.1371/journal.pone.0234627 (PMC7292398; doi:10.1371/journal.pone.0234627)
Supplement: S1 Table — “+” and “—” indicate presence or absence of data. (DOCX) [file pone.0234627.s002.docx]

**Table S1. *Cuscuta* species previously studied for seed morphology and/or anatomy arranged alphabetically and indicating their publication source. “+” and “—” indicate presence or absence of data.**

| ***Cuscuta* species** | **Morphology** | **Anatomy** | **Literature** |
| --- | --- | --- | --- |
| *C. australis* | + | — | Knepper et al. (1990) |
|  | — | + | Jayasuriya et al. (2008) |
| *C. brevistyla* | + | — | “*C. brevistylosa*”; Abdel-Khalik (2006) |
| *C. campestris* | — | + | Govil and Lavania (1980) |
|  | + | + | Gaertner (1950) |
|  | + | + | Hutchinson and Ashton (1979) |
|  | + | + | Lyshede (1984, 1992) |
|  | + | — | Abdel Khalik et al. (2006) |
|  | — | + | Costea et al. (2006) |
| *C. cephalanthi* | + | + | Gaertner (1950) |
| *C. chinensis* | + | — | (var. *chinensis*) Knepper et al. (1990) |
|  | + | — | Abdel-Khalik et al. (2006) |
| *C. compacta* | + | — | Knepper et al. (1990) |
| *C. epilinum* | + | + | Gaertner (1950) |
|  | + | — | Abdel-Khalik et al. (2006) |
| *C. epithymum* | + | + | Gaertner (1950) |
| *C. europaea* |  | + | Govil and Lavania (1980) |
|  | — | + | Martinčová et al. (2019) |
| *C. glomerata* | + | + | Gaertner (1950) |
| *C. gronovii* | + | + | Gaertner (1950) |
|  | + | — | Knepper et al. (1990) |
| *C. hyalina* |  | + | Tiagi (1966) |
| *C. indecora* | + | + | Gaertner (1950) |
| *C. japonica* | + | — | Knepper et al. (1990) |
|  | + | + | Costea et al. (2019) |
| *C. monogyna* | + | — | Knepper et al. (1990) |
|  | + | — | Abdel-Khalik et al. (2006) |
|  | — | + | Martinčová et al. (2019) |
| *C. obtusiflora* | + | + | Gaertner (1950) |
|  | — | + | Rodriguez-Pontes (2009) |
| *C. pacifica* | — | + | Costea et al. (2006) |
| *C. palaestina* | + | — | Abdel-Khalik et al. (2006) |
| *C. pedicellata* | — | + | Guttenberg (1909) |
|  | — | + | Kamensky (1928) |
|  | + | + | Lyshede 1984, 1992 |
|  | + | — | Abdel-Khalik et al. (2006) |
| *C. pentagona* | + | — | Knepper et al. et al. (2006) |
| *C. planiflora* | + | + | Gaertner (1950) |
|  | + | — | Abdel-Khalik et al. (2006) |
| *C. reflexa* | — | + | Johri and Tiagi (1952) |

**References**

Abdel Khalik KN. Seed morphology of *Cuscuta* L. (Convolvulaceae) in Egypt and its systematic significance. Feddes Repert.2006; 117: 217–224.

Costea M, Stefanović S, García MA, De La Cruz S, Casazza ML, Green AJ. Waterfowl endozoochory: An overlooked long‐distance dispersal mode for *Cuscuta* (dodder). Am J Bot. 2016; 103: 957–962.

Costea M, El Miari H, Laczkó L, Fekete R, Molnár AV, Lovas-Kiss Á, Green AJ. The effect of gut passage by waterbirds on the seed coat and pericarp of diaspores lacking “external flesh”: Evidence for widespread adaptation to endozoochory in angiosperms. PLoS One. 2019; 14(12).

Gaertner EE. Studies of seed germination, seed identification, and host relationships in dodders, *Cuscuta* spp. Mem Cornell Univ Agri Exp Station. 1950; 294: 1–56.

Govil CM, Lavania S. Floral anatomy and embryology of some species of *Cuscuta L.* Proc. Indian Acad Sci. 1980; 89: 219–228.

Guttenberg H. Uber die anatomische Unterscheidung der Samen einiger *Cuscuta*-Arten - Naturwiss. Zeitschr. Forst-Landw. 1909; 7: 32–43.

Jayasuriya KM, Baskin JM, Geneve RL, Baskin CC, Chien CT. Physical dormancy in seeds of the holoparasitic angiosperm *Cuscuta australis* (Convolvulaceae, Cuscuteae): Dormancy-breaking requirements, anatomy of the water gap and sensitivity. Ann Bot. 2008; 102: 39–48.

Johri BM, Tiagi B. Floral morphology and seed formation in *Cuscuta reflexa*. Phytomorphology. 1952; 2: 162–180.

Kamensky KW. Das Stemulieren der Samenaufkeimung Einwirkung von kochendem Wasser und Wasser, falsche Keimung/Essais Semences. 1928. 5: 1–19.

Knepper DA, Creager RA, Mussleman LJ. Identifying dodder seed as contaminants in seed shipments. Seed Sci Technol. 1990; 18: 731–741.

Martinčová M, Kaštier P, Krasylenko YA, Gajdoš P, Čertík M, Matušíková I, Blehová A. Species-specific differences in architecture and chemical composition of dodder seeds. Flora. 2019; 256: 61–68.

Rodriguez-Pontes M. Seed formation and pollination system in *Cuscuta obtusiflora*: First record of preanthesis cleistogamy in parasitic plants and some functional inferences. Flora. 2009; 204: 228–237.

Tiagi B. A contribution to the morphology and embryology of *Cuscuta hyalina* Roth and *C. plantiflora* Tenore. Phytomorphology. 1966; 1: 9–21.
