# Supplementary material for: Cuscuta seeds: Diversity and evolution, value for systematics/identification and exploration of allometric relationships
Source: PLoS One. 2020 Jun 12;15(6):e0234627. doi: 10.1371/journal.pone.0234627 (PMC7292398; doi:10.1371/journal.pone.0234627)
Supplement: S2 Table — Refer to Table 1 for the character states. CP = Compression; S = Shape; ET = Embryo type; HP = Hilum position; HC = Hilum compression; Dep = Dry seed epidermis; Hep = Hydrated seed epidermis; ECS = Epidermal cell shape; OP = Presence of outer palisade layer; #C = number of embryo coils; L = Seed length (μm); W = Seed width (μm); ST = Seed thickness (μm); Hl = Hilum length (μm), HW = Hilum Width (μm); FL = Length of funicular scar (μm); ECD = Epidermal cell diameter (μm); EPT = Epidermal cell thickness (μm); Epidermal cell width (μm). (DOCX) [file pone.0234627.s003.docx]

**Table S2.** Seed character dataset for *Cuscuta*. Refer to Table 1 for the description of character states. CP = Compression; S = Shape; ET = Embryo type; HP = Hilum position; HC = Hilum compression; Dep = Dry seed epidermis; Hep = Hydrated seed epidermis; ECS = Epidermal cell shape; OP = Presence of outer palisade layer; #C = number of embryo coils; L = Seed length (μm); W = Seed width (μm); ST = Seed thickness (μm); Hl = Hilum length (μm), HW = Hilum Width (μm); FL = Length of funicular scar (μm); ECD = Epidermal cell diameter (μm); EPT = Epidermal cell thickness (μm); Epidermal cell width (μm).

| **Subg. GRAMMICA**  **CLADE A** - *Californicae* | CP | S | ET | HP | HC | DEp | HEP | ECS | OP | #C | L | W | ST | HL | HW | FL | ECD | ECT | ECW | OPT | IPT |
| --- | --- | --- | --- | --- | --- | --- | --- | --- | --- | --- | --- | --- | --- | --- | --- | --- | --- | --- | --- | --- | --- |
| *C. brachycalyx* | 1&2 | 1&3 | 2 | 1 | 1 | 1 | 1 | 2 | 1 | 2 | 1075.2 | 1019.8 | 707.56 | 154.47 | 138.73 | 57.98 | 37.517 | 53.12 | 31.676 | 23.84 | 80.172 |
| *C. occidentalis* | 1&2&3 | 1&5 | 2 | 1&2 | 1&2 | 1 | 1 | 2 | 1 | 2.5 | 1025.6 | 913.9 | 637.26 | 143.29 | 114.43 | 49.04 | 31.065 | 39.6 | 29.327 | 22.569 | 74.706 |
| *C. californica* | 1&2 | 1 | 2 | 1&2 | 1 | 1 | 1 | 2 | 1 | 2 | 1088 | 1042.3 | 712.23 | 167.01 | 146.41 | 60.7 | 41.002 | 44.79 | 36.126 | 21.901 | 68.083 |
| *C. salina* | 1&2&3 | 1&4 | 2 | 1&2 | 1 | 1 | 1 | 2 | 1 | 2 | 1022 | 984.03 | 684.43 | 182.81 | 141.37 | 61.58 | 41.55 | 37.48 | 30.538 | 24.223 | 49.008 |
| *C. pacifica* | 1&2&3 | 1&4 | 2 | 1&2 | 1 | 1 | 1 | 2 | 1 | 3.5 | 1367.1 | 1260.6 | 998.09 | 205.4 | 129.7 | 79.74 | 34.426 | 47.54 | 32.002 | 22.409 | 60.011 |
| *C. subinclusa* | 1 | 1 | 2 | 1&2 | 1 | 1 | 1 | 2 | 1 | 3 | 1571.5 | 1410.2 | 820.06 | 211.36 | 196.26 | 91.23 | 32.333 | 40.2 | 29.866 | 26.556 | 75.943 |
| *C. suksdorfii* | 2 | 1 | 2 | 1&2 | 1 | 1 | 1 | 2 | 1 | 3 | 999.75 | 978.35 | 725.1 | 249.7 | 217.6 | 81.91 | 44.046 | 53.8 | 35.875 | 26.36 | 60.305 |
| *C. howelliana* | 1&2 | 1 | 2 | 1 | 1 | 1 | 1 | 2 | 1 | 2.5 | 910.05 | 747.9 | 479.5 | 94.27 | 87.97 | 40.71 | 43.911 | 21.76 | 17.96 | 22.3 | 48.095 |
| *C. draconella* | 1&3 | 1 | 2 | 1&2 | 1 | 1 | 1 | 2 | 1 | 2.5 | 1237.1 | 1158.3 | 768.83 | 184.9 | 141.3 | 62.34 | 40.929 | 37.6 | 27.809 | 16.133 | 52.103 |
| **CLADE B** *- Cleistogrammica* |  |  |  |  |  |  |  |  |  |  |  |  |  |  |  |  |  |  |  |  |  |
| *C. obtusiflora* var. *glandulosa* | 1&2&3 | 1&4 | 2 | 1&2 | 1 | 1 | 1 | 2 | 1 | 2.5 | 1367.6 | 1337.4 | 919.53 | 283.25 | 240.88 | 92.05 | 35.401 | 47.9 | 31.795 | 25.87 | 91.786 |
| *C. australis* var. *australis* | 1&2 | 1&4 | 2 | 1 | 1 | 1 | 1 | 2 | 1 | 2.5 | 1270.6 | 1223.2 | 777.32 | 214.33 | 231.11 | 98.29 | 28.085 | 36.2 | 26.618 | 22.808 | 89.183 |
| *C. campestris* | 2 | 1 | 2 | 1 | 1 | 1 | 1 | 2 | 1 | 2.5 | 1273 | 1133.8 | 884.33 | 244.2 | 207.1 | 77.65 | 36.271 | 45.2 | 32.666 | 25.287 | 89.976 |
| *C. pentagona* | 1 | 1 | 2 | 1&2 | 1 | 1 | 1 | 2 | 1 | 2.5 | 1159.4 | 1135.6 | 652.92 | 224.66 | 170.83 | 74.74 | 28.299 | 36.9 | 23.66 | 24.349 | 77.91 |
| *C. harperi* | 1&3 | 1 | 2 | 1&2 | 1 | 1 | 1 | 2 | 1 | 2.5 | 973.27 | 1034.8 | 630.47 | 188.2 | 147.05 | 63.1 | 25.021 | 26.9 | 20.348 | 21.557 | 76.722 |
| *C. glabrior* | 1 | 1 | 2 | 1&2 | 1 | 1 | 1 | 2 | 1 | 2.5 | 1396.6 | 1388.8 | 984.61 | 228.25 | 184.6 | 98.16 | 31.872 | 38.78 | 30.919 | 26.967 | 97.266 |
| *C. stenolepis* | 1 | 1&4 | 2 | 1 | 1&2 | 1 | 1 | 2 | 1 | 3 | 1261.3 | 1310 | 671.1 | 192 | 153.2 | 90.28 | 32.45 | 25.9 | 19.6 | 24.418 | 59.551 |
| *C. sandwichiana* | 1&2 | 1 | 2 | 2 | 1 | 1 | 1 | 2 | 1 | 3 | 1640.3 | 1408.7 | 1181.1 | 312.96 | 245.73 | 98.73 | 37.7765 | 76.37 | 43.062 | 37.164 | 85.731 |
| *C. polygonorum* | 1&2 | 1&4 | 2 | 2 | 1 | 1 | 1 | 2 | 1 | 3 | 1132.8 | 1156.7 | 819.21 | 237.93 | 183.81 | 98.35 | 29.954 | 41.98 | 24.968 | 25.624 | 71.325 |
| **CLADE C** *-* *Racemosae* |  |  |  |  |  |  |  |  |  |  |  |  |  |  |  |  |  |  |  |  |  |
| *C. incurvata* | 1 | 1 | 2 | 1 | 1 | 1 | 1 | 2 | 1 | 3 | 1311 | 1284 | 1044 | 171.8 | 122.4 | 63.04 | 18.4 | 22.8 | 19.03 | 23.56 | 84.6 |
| *C. xanthochortos* var. *carinata* | 1 | 1 | 2 | 1 | 1 | 1 | 1 | 2 | 1 | 2.5 | 1323.5 | 1756 | 1447 | 204.1 | 215.7 | 72.76 | 33.57 | 28.5 | 20.91 | 17.54 | 81.96 |
| *C. suaveolens* | 2 | 5 | 2 | 1 | 1 | 1 | 1 | 2 | 0 | 2.5 | 1454 | 1112 | 1086 | 193.3 | 155.1 | 105.2 | 31.7 | 22.7 | 17.8 | 19.83 | 76.85 |
| *C. platyloba* | 1 | 1 | 2 | 2 | 2 | 1 | 1 | 2 | 1 | 3 | 1503.5 | 1622 | 1423 | 219.5 | 162.4 | 111.4 | 27 | 23.71 | 14.83 | 23.24 | 62.72 |
| **CLADE D** *-* *Oxycarpae* |  |  |  |  |  |  |  |  |  |  |  |  |  |  |  |  |  |  |  |  |  |
| *C. cuspidata* | 1&2 | 1&3&5 | 2 | 1&2 | 1 | 1 | 1 | 2 | 1 | 2.5 | 1515.9 | 1465.8 | 975.88 | 305.38 | 227.61 | 118.7 | 30.285 | 43.2 | 30.779 | 24.513 | 97.689 |
| *C. squamata* | 1&2 | 1&4 | 2 | 2 | 1 | 1 | 1 | 2 | 1 | 2.5 | 1401.2 | 1187 | 742.71 | 222.95 | 177.4 | 62.64 | 43.636 | 41.19 | 33.88 | 23.095 | 77.153 |
| *C. compacta* | 1 | 1&4&5 | 2 | 1 | 1 | 1 | 1 | 2 | 1 | 2.5 | 1981.4 | 1790.9 | 1201.7 | 409.44 | 268.12 | 177.4 | 40.548 | 47.73 | 47.625 | 34.994 | 106.09 |
| *C. rostrata* | 2 | 1 | 2 | 1&2 | 1 | 1 | 1 | 2 | 1 | 3 | 1860.7 | 1700.3 | 1044.8 | 429.94 | 317.99 | 211.8 | 42.338 | 58.63 | 32.496 | 30.923 | 92.959 |
| *C. gronovii* var. *gronovii* | 2 | 1 | 2 | 2 | 1 | 1 | 1 | 2 | 1 | 3 | 1767 | 1578.8 | 1275.4 | 387.74 | 355.62 | 156.6 | 58.45 | 42.8 | 41.909 | 31.678 | 101.13 |
| *C. cephalanthi* | 1&2 | 5 | 2 | 1 | 1 | 1 | 1 | 2 | 1 | 3 | 1345.3 | 1294.5 | 977.58 | 254.85 | 238.46 | 87.21 | 35.976 | 41.88 | 32.472 | 25.101 | 89.007 |
| *C. umbrosa* | 1 | 1 | 2 | 2 | 1 | 1 | 1 | 2 | 1 | 3 | 1984.5 | 1900.9 | 1180.6 | 352.07 | 356.13 | 179.2 | 41.615 | 38.88 | 36.131 | 35.327 | 114.94 |
| *C. glomerata* | 1&2 | 5 | 2 | 1 | 1 | 1 | 1 | 2 | 1 | 2.5 | 1251.1 | 1075.7 | 816.85 | 249.98 | 223.36 | 73.41 | 32.083 | 36.7 | 30.64 | 23.674 | 86.947 |
| **CLADE E** *- Denticulatae* |  |  |  |  |  |  |  |  |  |  |  |  |  |  |  |  |  |  |  |  |  |
| *C. denticulata* | 1&2 | 3&4 | 1 | 1 | 1 | 1 | 1 | 2 | 0 | 1.5 | 811.69 | 698.95 | 539.31 | 143.67 | 132.1 | 39.51 | 26.989 | 43.9 | 20.024 | 18.73 | 42.27 |
| *C. veatchii* | 3 | 1 | 1 | 1 | 1&2 | 1 | 1 | 2 | 0 | 0.5 | 1001.4 | 887.57 | 655.39 | 169.75 | 179.35 | 48.75 | 50.979 | 26.67 | 28.805 | 19.59 | 43.912 |
| *C. nevadensis* | 3 | 1&4 | 1 | 1 | 1 | 1 | 1 | 2 | 0 | 0.5 | 998.81 | 990.78 | 847.02 | 182.96 | 144.26 | 61.63 | 43.59 | 35.23 | 25.415 | 19.43 | 43.71 |
| **CLADE F** *-* *Partitae* |  |  |  |  |  |  |  |  |  |  |  |  |  |  |  |  |  |  |  |  |  |
| *C. haughtii* | 1&2 | 1&5 | 2 | 1&2 | 1 | 1 | 1 | 2 | 1 | 2.5 | 945.88 | 1532.9 | 561.13 | 184.06 | 150.96 | 64.1 | 26.406 | 30.26 | 19.539 | 19.841 | 69.078 |
| *C. partita* | 1&2 | 1 | 2 | 1&2 | 1 | 1 | 1 | 2 | 1 | 2 | 1181.6 | 1018.2 | 815.3 | 172.5 | 138.33 | 64.42 | 26.2 | 26.99 | 25.996 | 23.696 | 76.75 |
| **CLADE G** *-* *Lobostigmae* |  |  |  |  |  |  |  |  |  |  |  |  |  |  |  |  |  |  |  |  |  |
| *C. tinctoria var. floribunda* | 1&2 | 4&5 | 2 | 1&2 | 1 | 1 | 1 | 2 | 1 | 2 | 1472.1 | 1282.8 | 849.85 | 241.14 | 186.1 | 85.95 | 36.913 | 61.85 | 32.671 | 25.306 | 94.457 |
| *C. mitriformis* | 1&2 | 1&5 | 2 | 1&2 | 1 | 1 | 1 | 2 | 1 | 3.5 | 2020.9 | 1640.6 | 1138.7 | 357.86 | 300.73 | 139.2 | 44.005 | 51.096 | 47.99 | 34.808 | 123.35 |
| *C. jalapensis* | 1&2 | 1&4 | 2 | 1&2 | 1 | 1 | 1 | 2 | 1 | 3.5 | 1698.5 | 1700.4 | 957.68 | 340.75 | 301.04 | 90.83 | 45.956 | 55.9 | 46.272 | 35.166 | 101.4 |
| *C. rugosiceps* | 1&2 | 1&4 | 2 | 2 | 1 | 1 | 1 | 2 | 1 | 3 | 1791.6 | 1405.2 | 1171.7 | 279.73 | 238.66 | 94.7 | 39.966 | 73.87 | 46.763 | 44.272 | 114.7 |
| *C. woodsonii* | 2 | 1 | 2 | 2 | 1 | 1 | 1 | 2 | 1 | 3 | 2026.8 | 1954.4 | 1200.9 | 306.74 | 300.2 | 139.8 | 53.23 | 49.1 | 56.234 | 40.21 | 143.2 |
| *C. volcanica* | 2 | 1&5 | 2 | 1&2 | 1 | 1 | 1 | 2 | 1 | 2.5 | 2124.9 | 1659.3 | 973.5 | 522.65 | 400.85 | 147.9 | 50.06 | 116.5 | 40.305 | 33.917 | 113.05 |
| *C. tasmanica* | 1&2 | 2 | 2 | 2 | 1 | 1 | 1 | 2 | 1 | 2.5 | 1565.5 | 1378.8 | 875.58 | 260.42 | 216.22 | 86.5 | 36.704 | 76.37 | 30.128 | 27.508 | 97.62 |
| *C. iguanella* | 1&3 | 1&4&5 | 2 | 1&2 | 1 | 1 | 1 | 2 | 1 | 3 | 1386.1 | 1104.7 | 749.04 | 285.87 | 243.99 | 95.61 | 32.967 | 44.24 | 35.836 | 18.161 | 84.785 |
| *C. montana* | 1&3 | 4 | 2 | 1&2 | 2 | 1 | 1 | 2 | 1 | 2.5 | 1588.7 | 1253.7 | 929.1 | 248.8 | 223.9 | 130.2 | 41.23 | 56.99 | 38.018 | 28.93 | 82.62 |
| *C. timida* | 2 | 2&3 | 2 | 1&2 | 1 | 1 | 1 | 2 | 1 | 3 | 1414.8 | 1272.7 | 907.15 | 280.96 | 203.53 | 93.29 | 38.903 | 54.65 | 27.62 | 25.63 | 56.196 |
| *C. purpusii* | 1 | 1 | 2 | 1&2 | 1 | 1 | 1 | 2 | 1 | 3 | 1574.7 | 1318.7 | 939.59 | 264.21 | 208.12 | 95.56 | 42.204 | 32.3 | 37.111 | 28.607 | 94.344 |
| *C. victoriana* | 1&2 | 1 | 2 | 1&2 | 1 | 1 | 1 | 2 | 1 | 3.5 | 1231.2 | 1069.4 | 801.58 | 221.68 | 209.23 | 74.87 | 42.773 | 51.92 | 34.29 | 21.632 | 60.301 |
| **CLADE H** *-* *Obtusilobae* |  |  |  |  |  |  |  |  |  |  |  |  |  |  |  |  |  |  |  |  |  |
| *C. macrocephala* | 1&2 | 1&3&4 | 2 | 1&2 | 1 | 1 | 1 | 2 | 1 | 3 | 1469.5 | 1368.7 | 848.74 | 168.96 | 146.38 | 80.11 | 33.318 | 60.28 | 36.613 | 27.282 | 93.38 |
| *C. globulosa* | 1 | 3 | 2 | 1&2 | 1 | 1 | 1 | 2 | 1 | 2.5 | 1337.6 | 1280.1 | 876.56 | 247.89 | 257.38 | 76.18 | 29.773 | 52.7 | 28.997 | 23.725 | 94.905 |
| *C. americana* | 1&2 | 1&3&4 | 2 | 1 | 1 | 1 | 1 | 2 | 1 | 2 | 1294.9 | 1157.4 | 851.61 | 228.32 | 218.13 | 60.99 | 39.384 | 40.69 | 35.92 | 24.816 | 81.804 |
| **CLADE I** *-* *Grammica* |  |  |  |  |  |  |  |  |  |  |  |  |  |  |  |  |  |  |  |  |  |
| *C. azteca* | 1&2 | 1&3&5 | 2 | 1 | 1 | 1 | 1 | 2 | 1 | 2.5 | 1065.5 | 1024.1 | 723.51 | 194.35 | 175.12 | 65.7 | 26.335 | 28.2 | 25.979 | 24.38 | 76.441 |
| *C. yucatana* | 1&2&3 | 1 | 2 | 2 | 1 | 1 | 1 | 2 | 1 | 2 | 1030.2 | 921.3 | 695.72 | 199.51 | 157.06 | 65.95 | 30.201 | 27.115 | 25.56 | 24.789 | 68.478 |
| *C. alata* | 1&2 | 1&4 | 2 | 1 | 1 | 1 | 1 | 2 | 1 | 2.5 | 943.4 | 832.37 | 708.74 | 171.02 | 154.35 | 70.43 | 30.702 | 41.73 | 24.452 | 18.142 | 69.71 |
| **CLADE J** *-* *Prismaticae* |  |  |  |  |  |  |  |  |  |  |  |  |  |  |  |  |  |  |  |  |  |
| *C. corymbosa* var. *grandiflora* | 1&2 | 1 | 2 | 1 | 1&2 | 1 | 1 | 2 | 1 | 2 | 1669.9 | 1497.6 | 817.66 | 221.8 | 187.97 | 81.32 | 25.998 | 37.57 | 29.12 | 30.413 | 103.4 |
| *C. corymbosa* var. *stylosa* | 1&2 | 1 | 2 | 1 | 1 | 1 | 1 | 2 | 1 | 2 | 1127.4 | 992.41 | 585.65 | 179.78 | 138.69 | 60.05 | 24.445 | 40.081 | 22.713 | 20.657 | 61.54 |
| **CLADE K** *-* *Ceratophorae* |  |  |  |  |  |  |  |  |  |  |  |  |  |  |  |  |  |  |  |  |  |
| *C. chapalana* | 1&2&3 | 5 | 2 | 1 | 1 | 1 | 1 | 2 | 1 | 2 | 1661.3 | 1309.1 | 835.4 | 230.48 | 192.73 | 86.99 | 40.351 | 41.03 | 37.768 | 33.46 | 102.83 |
| *C. strobilacea* var. *pringlei* | 2 | 4 | 2 | 1&2 | 1 | 1 | 1 | 2 | 1 | 2 | 1523.9 | 1500 | 858.89 | 270.05 | 203.83 | 86.5 | 35.348 | 53.44 | 28.581 | 25.274 | 63.953 |
| *C. erosa* | 2 | 1&4 | 2 | 1&2 | 1 | 1 | 1 | 2 | 1 | 2.5 | 1217.4 | 1031.3 | 704.02 | 216.48 | 173.85 | 82.67 | 31.977 | 45.4 | 33.594 | 17.445 | 94.55 |
| *C. boldinghii* | 2 | 3&4 | 2 | 1 | 1 | 1 | 1 | 2 | 1 | 2 | 981.35 | 857.96 | 637.23 | 162.8 | 168.05 | 66.05 | 33.97 | 52.34 | 32.534 | 24.493 | 82.85 |
| *C. costaricensis* | 1 | 1 | 2 | 2 | 1 | 1 | 1 | 2 | 1 | 2.5 | 1144 | 1032.9 | 720.45 | 257.7 | 196.95 | 83.88 | 44.686 | 39.56 | 32.348 | 23.405 | 79.44 |
| *C. bonafortunae* | 2 | 5 | 2 | 2 | 1 | 1 | 1 | 2 | 1 | 2.5 | 1363.7 | 1029.5 | 742.65 | 236.9 | 191.95 | 53.06 | 40.541 | 51.58 | 26.818 | 26.485 | 132.4 |
| **CLADE L** *-* *Umbellatae* |  |  |  |  |  |  |  |  |  |  |  |  |  |  |  |  |  |  |  |  |  |
| *C. odontolepis* | 2&3 | 1&5 | 2 | 1&2 | 1 | 1 | 1 | 2 | 1 | 3 | 1109 | 856.53 | 862.9 | 165.4 | 164.7 | 66.36 | 31.822 | 46.23 | 20.25 | 22.705 | 59.365 |
| *C. legitima* | 1&2 | 1&4&5 | 2 | 1&2 | 1 | 1 | 1 | 2 | 1 | 3 | 1001.3 | 813.4 | 600.63 | 169.05 | 147.54 | 52.59 | 27.374 | 25.98 | 24.969 | 18.444 | 64.046 |
| *C. tuberculata* | 1&2&3 | 1 | 2 | 1&2 | 1 | 1 | 1 | 2 | 1 | 2.5 | 886.43 | 759.39 | 528.38 | 168.2 | 136.22 | 66.88 | 23.386 | 38 | 19.377 | 13.842 | 45.736 |
| *C. umbellata* | 1&2&3 | 1&4 | 2 | 1&2 | 1 | 1 | 1 | 2 | 1 | 2.5 | 1064 | 973.13 | 671.29 | 194.4 | 176.13 | 55.55 | 27.057 | 32.6 | 26.946 | 18.382 | 56.662 |
| *C. desmouliniana* | 1&2 | 3&4&5 | 2 | 1&2 | 1 | 1 | 1 | 2 | 1 | 2 | 861.41 | 760.09 | 543.83 | 143.3 | 122.95 | 69.1 | 26.683 | 45.43 | 20.836 | 15.476 | 45.131 |
| *C. acuta* | 1&2 | 3&4&5 | 2 | 1 | 1 | 1 | 1 | 2 | 1 | 2 | 972.46 | 853.63 | 622.06 | 195.81 | 180.47 | 38.24 | 33.876 | 26.39 | 26.151 | 22.569 | 69.654 |
| *C. leptantha* | 1&2&3 | 1&3 | 2 | 1&2 | 1 | 1 | 1 | 2 | 1 | 2 | 784.7 | 679.96 | 505.91 | 116.86 | 99.915 | 32.06 | 22.013 | 29.91 | 26.429 | 14.759 | 54.785 |
| *C. polyanthemos* | 2 | 5 | 2 | 2 | 1 | 1 | 1 | 2 | 1 | 2.5 | 930.2 | 668.27 | 431.43 | 110.22 | 104.34 | 47.82 | 17.504 | 37.04 | 21.249 | 22.24 | 54.223 |
| *C. liliputana* | 1&2 | 1&4 | 2 | 2 | 1 | 1 | 1 | 2 | 1 | 2 | 939.2 | 848.57 | 522.6 | 136.34 | 104.36 | 76.64 | 25.272 | 41.08 | 16.613 | 18.741 | 62.132 |
| *C. membranacea* | 1&2 | 1&3&4&5 | 2 | 1&2 | 1 | 1 | 1 | 2 | 1 | 2 | 1075.8 | 958.33 | 600.31 | 197.06 | 180.03 | 72.66 | 35.115 | 20.61 | 28.576 | 23.758 | 66.824 |
| **CLADE M** - *Indecorae* |  |  |  |  |  |  |  |  |  |  |  |  |  |  |  |  |  |  |  |  |  |
| *C. warneri* | 1 | 1&5 | 2 | 2 | 1 | 1 | 1 | 2 | 1 | 2.5 | 1362.2 | 1599 | 911.95 | 238.25 | 171.3 | 78.23 | 41.026 | 39.4 | 29.083 | 28.07 | 69.77 |
| *C. coryli* | 1 | 3 | 2 | 2 | 1 | 1 | 1 | 2 | 1 | 2.5 | 1795 | 2262 | 1897 | 507.3 | 310.3 | 238.4 | 42.6 | 25.26 | 25.61 | 24.58 | 89.9 |
| *C. indecora* var. *indecora* | 1&3 | 1&5 | 2 | 1&2 | 1 | 1 | 1 | 2 | 1 | 2.5 | 1442.5 | 1544 | 800.56 | 298.61 | 208.36 | 66.25 | 40.926 | 37.81 | 35.477 | 35.906 | 113.32 |
| **CLADE N** - Gracillimae |  |  |  |  |  |  |  |  |  |  |  |  |  |  |  |  |  |  |  |  |  |
| *C. gracillima* | 1&2 | 3 |  | 2 | 1 | 1 | 1 | 2 | 1 | 2 | 1047.9 | 977.86 | 934.7 | 183.55 | 158.77 | 76.48 | 29.776 | 50.31 | 24.49 | 22.54 | 76.51 |
| *C. sidarum* | 3 | 1 | 2 | 1 | 1 | 1 | 1 | 2 | 1 | 2.5 | 814.5 | 752.85 | 501.5 | 142.25 | 119.05 | 51.44 | 34.778 | 48.48 | 13.61 | 18.7 | 53.815 |
| *C. vandevenderi* | 2&3 | 1 | 2 | 2 | 1 | 1 | 1 | 2 | 1 | 2 | 1034.2 | 967.32 | 564.9 | 166.13 | 169.73 | 72.07 | 33.938 | 42.96 | 24.8 | 22.544 | 61.89 |
| **CLADE O** - *Subulatae* |  |  |  |  |  |  |  |  |  |  |  |  |  |  |  |  |  |  |  |  |  |
| *C. odorata* var. *odorata* | 1&2 | 1&4&5 | 2 | 2 | 1 | 1 | 1 | 2 | 1 | 2 | 1347.6 | 1391.6 | 946.4 | 294.05 | 273.57 | 74.35 | 40.933 | 18.02 | 43.35 | 27.69 | 88.79 |
| *C. chilensis* | 1&2 | 1&5 | 2 | 1 | 1 | 1 | 1 | 2 | 1 | 2 | 1503.7 | 1374.8 | 867.46 | 249.53 | 213.44 | 99.98 | 40.163 | 38.8 | 55.506 | 26.473 | 93.596 |
| *C. purpurata* | 1&2 | 1&4&5 | 2 | 2 | 1 | 1 | 1 | 2 | 1 | 2 | 1610.6 | 1469.8 | 1053.7 | 281.65 | 237.94 | 80.12 | 48.917 | 30.36 | 46.066 | 33.235 | 116.14 |
| *C. foetida* var. *foetida* | 1&2 | 1&3&5 | 2 | 1&2 | 1 | 1 | 1 | 2 | 1 | 2.5 | 1502.6 | 1359.3 | 1234.2 | 315.36 | 274.93 | 143.1 | 42.141 | 48.82 | 43.672 | 34.714 | 103.31 |
| *C. grandiflora* | 1&2 | 1&2&3&4&5 | 2 | 2 | 1 | 1 | 1 | 2 | 1 | 3 | 1887.3 | 1776.9 | 972.65 | 329.87 | 286.57 | 100.1 | 50.546 | 92.04 | 54.589 | 36.952 | 96.065 |
| *C. parodiana* | 2 | 1&4 | 2 | 1&2 | 1 | 1 | 1 | 2 | 1 | 2 | 1455.2 | 1328 | 793.1 | 256.63 | 198.06 | 116.2 | 49.987 | 81.81 | 38.129 | 24.62 | 80.575 |
| *C. cristata* | 1&2 | 1&4&5 | 2 | 1 | 1 | 1 | 1 | 2 | 1 | 2.5 | 1439 | 1332.2 | 1066.2 | 363.06 | 290.32 | 90.34 | 36.637 | 66.76 | 34.586 | 25.85 | 96.772 |
| *C. microstyla* | 3 | 3 | 1 | 1 | 2 | 1 | 1 | 2 | 1 | 1 | 1193.1 | 1050.5 | 831.93 | 192.4 | 124.6 | 71.5 | 55.132 | 66.3 | 44.411 | 18.36 | 35.91 |
| *C. acutiloba* | 1&2 | 1&3 | 2 | 1 | 1 | 1 | 1 | 2 | 1 | 2.5 | 900.25 | 818.42 | 714.31 | 140.7 | 133.56 | 114.4 | 35.29 | 46.5 | 34.816 | 17.115 | 81.253 |
| *C. boliviana* | 1&2&3 | 1&4&5 | 2 | 1 | 1&2 | 1 | 1 | 2 | 1 | 2.5 | 1769 | 1672.9 | 1143.1 | 323.93 | 282.44 | 54.06 | 55.969 | 43.6 | 55.5 | 33.402 | 110.41 |
| *C. paitana* | 1&3 | 1 | 2 | 1 | 1 | 1 | 1 | 2 | 1 | 3 | 704.55 | 1021 | 519.7 | 134.4 | 114 | 60.13 | 24.894 | 51.2 | 17.662 | 10.4 | 51.18 |
| *C. goyaziana* | 1 | 1 | 2 | 2 | 1 | 1 | 1 | 2 | 1 | 3 | 1343.7 | 1313.5 | 576.7 | 259 | 249.6 | 65.45 | 44.345 | 40.5 | 39.616 | 19.7 | 75.715 |
| **Subg. PACHYSTIGMA** |  |  |  |  |  |  |  |  |  |  |  |  |  |  |  |  |  |  |  |  |  |
| *C. nitida* | 1 | 1 | 2 | 2 | 1 | 1 | 1 | 2 | 1 | 1.5 | 1072.2 | 881.15 | 679.27 | 231.9 | 202.3 | 58.32 | 33.218 | 26.08 | 29.355 | 16.602 | 67.975 |
| **Subg. CUSCUTA** |  |  |  |  |  |  |  |  |  |  |  |  |  |  |  |  |  |  |  |  |  |
| *C. planiflora* | 1&2 | 1 | 2 | 2 | 1 | 1 | 1 | 2 | 1 | 2 | 1052.7 | 852 | 627.03 | 199.36 | 163.5 | 103.5 | 48.493 | 58.42 | 35.624 | 22.4 | 69.78 |
| *C. europaea* | 1&2 | 1&4&5 | 2 | 1&2 | 1 | 1 | 1 | 2 | 1 | 2 | 1277.6 | 1101.8 | 906.82 | 284.73 | 238.71 | 103.4 | 42.215 | 50.25 | 35.617 | 20.045 | 59.565 |
| *C. epilinum* | 1&2 | 1&3&4&5 | 2 | 1&2 | 1 | 1 | 1 | 2 | 1 | 2 | 1241.6 | 1076.8 | 811.89 | 282.89 | 226.21 | 53.67 | 49.822 | 67.17 | 40.874 | 24.857 | 58.7 |
| *C. approximata* | 2&3 | 1 | 2 | 1 | 1 | 1 | 1 | 2 | 1 | 2 | 1096.5 | 858.24 | 781.13 | 167.08 | 166.62 | 56.85 | 40.206 | 57.39 | 34.305 | 20.66 | 70.208 |
| *C. epithymum* | 1&2 | 1&4&5 | 2 | 1&2 | 1 | 1 | 1 | 2 | 1 | 2 | 896.28 | 754.46 | 615.51 | 171.4 | 142.13 | 52.11 | 38.044 | 54.54 | 28.687 | 16.334 | 54.13 |
| *C. babylonica* | 2 | 1&3 | 2 | 1 | 1&2 | 1 | 1 | 2 | 1 | 2 | 839.38 | 744.34 | 506.52 | 142 | 136.15 | 48.69 | 38.468 | 79.83 | 34.836 | 18.618 | 49.977 |
| **Subg.** MONOGYNELLA |  |  |  |  |  |  |  |  |  |  |  |  |  |  |  |  |  |  |  |  |  |
| *C. monogyna* | 1 | 4 | 2 | 1 | 1&2 | 0 | 0 | 1 | 0 | 1 | 2533.91 | 2151.4 | 1479.5 | 488.77 | 359.08 | 575.3 | - | 49.94 | 23.832 | 34.56 | 111.72 |
| *C. japonica* | 1&2&3 | 1&5 | 2 | 1 | 1 | 0 | 0 | 1 | 0 | 2 | 2457.4 | 2078.5 | 1746.45 | 715.76 | 520.66 | 539.3 | - | 29.89 | 28.939 | 45.37 | 150.48 |
| *C. lupuliformis* | 3 | 4&5 | 2 | 1 | 1 | 0 | 0 | 1 | 0 | 2 | 2656 | 2389.5 | 1820.5 | 647.6 | 659.9 | 454.06 | - | 26.37 | 26.28 | 60.505 | 108.54 |
| *C. reflexa* | 1&2 | 1&4 | 2 | 1&2 | 1 | 0 | 0 | 1 | 0 | 1 | 3158.3 | 2910 | 2133.16 | 774.91 | 505.13 | 506.2 | - | 22.96 | 36.453 | 41.305 | 162.99 |
| *C. gigantea* | 1&2 | 1&5 | 2 | 2 | 1 | 0 | 0 | 1 | 0 | 2 | 2860 | 2434 | 1488.5 | 811.25 | 637.4 | 496.1 | - | 34.22 | 30.128 | 42.215 | 111.75 |
