# Supplementary material for: Cuscuta seeds: Diversity and evolution, value for systematics/identification and exploration of allometric relationships
Source: PLoS One. 2020 Jun 12;15(6):e0234627. doi: 10.1371/journal.pone.0234627 (PMC7292398; doi:10.1371/journal.pone.0234627)
Supplement: S3 Table — (DOCX) [file pone.0234627.s004.docx]

**Table S3. Summary of basic statistics for quantitative characters of Cuscuta seeds.**

| **Seed trait (µm)** | **Mean** | **Median** | **Std. Error** | **Min** | **Max** | **p-value (Shapiro)** |
| --- | --- | --- | --- | --- | --- | --- |
| Seed length | 1353.5 | 1265.9 | 45.1 | 704.55 | 3158.3 | 8.877E-08 |
| Seed width | 1235.3 | 1156.2 | 40.9 | 668.28 | 2910.05 | 2.178E-06 |
| Seed thickness | 842.3 | 806.7 | 29.2 | 388.4 | 2133.2 | 7.212E-07 |
| Hilum area length | 256.5 | 228.3 | 13.2 | 94.3 | 811.3 | 3.323E-11 |
| Hilum area width | 215.4 | 192.7 | 10.2 | 97.97 | 659.9 | 1.464E-10 |
| Length of funicular scar of the hilum | 99 | 77.7 | 9.7 | 32.1 | 575.4 | 5.46E-16 |
| Epidermal cell thickness | 45.7 | 43.4 | 1.7 | 18.0 | 116.6 | 2.374E-06 |
| Epidermal cell width | 32.4 | 30.9 | 0.92 | 13.6 | 56.2 | 0.006912 |
| Outer palisade layer thickness | 25.6 | 24.4 | 0.8 | 10.4 | 60.5 | 7.586E-06 |
| Inner palisade layer thickness | 81.6 | 77.9 | 2.5 | 42.3 | 163 | 0.003854 |
