# Supplementary material for: Cuscuta seeds: Diversity and evolution, value for systematics/identification and exploration of allometric relationships
Source: PLoS One. 2020 Jun 12;15(6):e0234627. doi: 10.1371/journal.pone.0234627 (PMC7292398; doi:10.1371/journal.pone.0234627)
Supplement: S4 Table — (DOCX) [file pone.0234627.s005.docx]

**Table S4.** **Pearson’s correlation summary for quantitative seed characters of Cuscuta.**

| **Variables** | **r** | **r^2^** | **p** |
| --- | --- | --- | --- |
| Seed length x seed width | 0.95136 | 0.90905 | 9.5162 E-46 |
| Seed length x seed thickness | 0.91537 | 0.8379 | 1.5724E-36 |
| Inner palisade layer x outer palisade layer | 0.79283 | 0.62858 | 0.03899 |
| Length of hilar pad x width of hilar pad | 0.96906 | 0.93907 | 1.6345E-52 |
| Length of hilar pad x length of funicular scar | 0.93074 | 0.86627 | 1.41E-37 |
| Seed size x epidermal cell diameter | 0.58388 | 0.34091 | 1.1346E-06 |
| Seed Size x epidermal cell thickness | 0.30262 | 0.091576 | 0.0048779 |
| Outer palisade layer x epidermal cell thickness | 0.53551 | 0.28677 | 1.5318E-07 |
| Distribution of subg. *Grammica* x seed size | 0.094412 | 0.0089136 | No relationship |
