# Supplementary material for: Cuscuta seeds: Diversity and evolution, value for systematics/identification and exploration of allometric relationships
Source: PLoS One. 2020 Jun 12;15(6):e0234627. doi: 10.1371/journal.pone.0234627 (PMC7292398; doi:10.1371/journal.pone.0234627)
Supplement: S5 Table — (DOCX) [file pone.0234627.s006.docx]

**Table S5.** **Seed size of three *Cuscuta* species studied and summary of statistical results.**

| **Species** | **Seed size;**  **Average length x width**  [mm] | **Average seed mass**  [mg] | **r** | **p** |
| --- | --- | --- | --- | --- |
| *C. epithymum* | 0.89 x 0.76 | 0.098 | 0.024* | 0.003 |
| *C. costaricensis* | 1.08 x 0.93 | 0.1726 | 0.088* | 0.050 |
| *C. campestris* | 1.26 x 1.0 | 0.2425 | 0.098* | 0.012 |
