# Supplementary material for: Cuscuta seeds: Diversity and evolution, value for systematics/identification and exploration of allometric relationships
Source: PLoS One. 2020 Jun 12;15(6):e0234627. doi: 10.1371/journal.pone.0234627 (PMC7292398; doi:10.1371/journal.pone.0234627)
Supplement: S1 Appendix — (DOCX) [file pone.0234627.s007.docx]

**Appendix S1.** List of herbarium specimens examined for seeds in *Cuscuta*. Species are arranged alphabetically. Country, locality details, date, collectors, and herbaria in which the specimens are deposited are provided for all specimens. Specimens used in the survival study are indicated with an asterisk. Abbreviations of herbaria follow Index Herbariorum.

***Cuscuta acuta*** Engelm.: **ECUADOR. Galapagos**, Chatam Isl., 1863, *Anderson 1853* (S); Tower Island, 16 Jun1932, *Howell 10140*(G); **Prov.** **Manabí**, Bahia de Caraquez, around Hotel La Herradura, 15 Feb1981, *Sparre 19700*(S). ***Cuscuta acutiloba*** Engelm.*:* **BOLIVIA. Prov. Larecaja**, vicinity of Sorata, May 1863, *Mandon 1481* (S). **PERU. Prov. Moquegua**, 22 Mar 1925, *Weberbauer 7443* (F); **Dept. Arequipa**, Arequipa, 7–16 Apr 1925, *Pennell 13242* (S). ***Cuscuta alata*** Brandegee: **MEXICO. Chihuahua**, South Western Chihuahua, Aug–Nov 1885, *Palmer 142* (GH). **Sinaloa**, Mpio. Culiacán, on Rd at Varejonal, 10 Km from the international Hwy, 12 Oct 1984, *Avalos & Bojórguez 196* (MEXU). ***Cuscuta americana*** L.: **MEXICO**. **Guerrero**, Acapulco and vicinity, Oct 1894, *Palmer 341* (GH). **Jalisco**, Mpio. La Huerta, near Chamela, 15 Apr 1982, *Lott 994* (MICH). **Sonora**, Mpio. Alamos, Arroyo el Mentidero at El Chinal Rd, 11.3 km S of Álamos, 26^o^54'45"N, 108^o^55'05"W, 15 Mar 1994, *Van Devender 94-176* (UCR). Mpio. Villa Pesqueira, southern foot of Sierra Pinta, ca. 29^o^35'N, 110^o^01'W, 15 Sep 1996, *Shortman 96-71* (ARIZ). **U.S.A. Florida**, Lower Matecumbe Key, 3 Jan 1925, *Small et al. 11596* (NY); Pinelands, Buena Vista, Miami, Jan 1930, *Mosier s.n*(NY). ***Cuscuta approximata*** Bab.: **CANADA. British Columbia**, Cherry Creek, Kamloops Lake, 30 Jul 1993, *Lomer 93-204* (UBC); Spences Bridge, 27 Sep 1992, *Lomer 92-306* (UBC). **U.S.A. California**, *Abrams 457* (CAS). **Utah**, Salt Lake City, 24 Sep 1905, *Jones s.n.* (RSA). ***Cuscuta australis*** R. Br. var. ***australis***: **AUSTRALIA. New South Wales**, Warrabah National Park, 30˚34’S, 150˚55’E, 13 Mar 1994, *Hosking 938* (CANB). **Queensland**, Maryborough, Wallum, 23 Oct 1948, *Clemens s.n* (RSA). **NEW CALEDONIA.** Nouméa, Feb 1907, *Bonati 737* (S). ***Cuscuta azteca*** Costea & Stefanović: **MEXICO. Federal District**, Pedrigal, Valley of Mexico, 8 Oct 1896, *Pringle 6575* (S). **Puebla**, 9 Jul 1908, *Purpus 3554* (MO). **Querétaro**, Mpio. Piñal de Amóles, 13.8 km SE MEX 120 on rd. to Bucareli, *Ramírez-Amezcua et al. 1099* (IEB). **San Luis Potosi**, Charcus, Jul-Aug 1934, *Lundell 5193* (S). ). ***Cuscuta boliviana*** Yunck. **ARGENTINA.** **Prov. Menza**, Depto. Las Heras, 7 Mar 1952, *Ruiz Leal 1954* (CTES) **Prov. Salta**, Depto. Molinos, Luracatao, 9 Apr 1982, Novara 2640 (CTES). **BOLIVIA.** Padcaya, 11 Dec 1903, *Fiebrig 2523* (G). ***Cuscuta boldinghii*** Urb.: **HAITI.** Massif de la Hotte, close to Dame Marie, 1 Aug 1928, *Ekman 10455* (S). **MEXICO. Jalisco**, 8 km E of Chamela, 8-10 Dec 1970, *McVaugh 25140* (MICH). ***Cuscuta bonafortunae*** Costea & I. García: **MEXICO. Guanajuato**, Santiago de Maravatío, close to Ojo de Agua, 1900 m, 15 Oct 1989, *Rzedowski 49127* (IEB). **Michoacán**, Zamora, 11 Sep 2010, *García Ruiz & García 8375* (CIMI, WLU); 16 Oct 2010, *García Ruiz 8391* (CIMI, WLU). ***Cuscuta brachycalyx*** (Yunck.) Yunck.: **U.S.A. California**, W of Lunt Rd., ca. 0.5 mile NW of Hwy 70, 10 Aug 1988, *Ahart 6170* (CAS); Kern Co., 1.5 mi. N of Kernville, 28 Sep 1962, *Howell 38877* (CAS). ***Cuscuta californica*** Hook. & Arn.: **U.S.A., California**, Colusa Co., Dark Hollow Creek, Snow Mt., 10 Sep 1974, *Heckard 3888* (JEPS); Los Angeles Co., ridge S of Swartout Valley, 31 Aug 1923, *Munz 7689* (RSA); Coastal Sage Scrub, Wash outside RSABG, 9 Oct1965, *Massey 1005* (SMU). ***Cuscuta campestris*** Yunck.: **U.S.A. California**, San Bernardino Co., North Verde Ranch, near Mojave River, E of Victorville, 10 Sep 1961, *Raven 16637* (RSA); San Bernardino Mts., Los Rios Rancho at Oak Glen Forest Falls, 11 Nov 1999, *Sanders 23249* (UCR); Sonoma Co., Rosa, 38°13'26.60’N, 122°49'42.10’W, Sep 2007, *Cadman et al*. *2832* (WLU)*. ***Cuscuta cephalanthi***Engelm.: **U.S.A. Indiana**, Newton Co., Near the Kankakee River, N of Lake Village, 22 Sep 1927,  *Deam 455*(NY); Sullivan Co., 6 mi SW of Carlisle, 4 Oct 1931, *Deam 51439*(IND). **Michigan**, Wayne Co., Oakwood, 15 Sep 1918, *Farwell 5124* (NY)*.***Minnesota**, St. Louis Co., shore of Sand Point Lake at Harrison’s Narrows international boundary, 11 Sep 1952, *Lakela 15846*(DAO)*.***Utah**, Salt Lake Co., Salt Lake City, 22 Aug 1880, *Jones s.n*(RSA). ***Cuscuta chapalana*** Yunck.: **MEXICO. Jalisco**, Mpio. Ojuelos de Jalisco: Rancho “Las Papas de Arriba”, 4.5 Km NE of Guadalupe Victoria, 21º43’48”N 101º39’48”W, 2260 m, 14 Oct 2000, *García & Harker 438* (IBUG); Mpio. Jocotepec: N face of Cerro Viejo, 2200 m, 17 Jan 1987, *Chazaro et al. 4408.* (IBUG); Barranca de Sayula, SE of San Pedro Tesistan, Nov1993, *Machuca 7026* (MICH). ***Cuscuta chilensis*** Ker Gawl: **ARGENTINA. Mendoza**, 1890-91, *Smith 1749* (S). **CHILE. Aconcagua**, Saladillo, 21 Apr 1973, *Castillo 4* (SGO), **Santiago**, Barnechea, hills near Nido de Aquilas school, 21 Jan 1978, *Landrum 3073* (ASU). **Valparaíso**, Uspallata-Pass, 16 Jan 1903, *Buchtien s.n.* (UPS). **U.S.A. *Cuscuta compacta*** Juss. ex. Choisy: **Alabama**, Dekalb Co., Westfork Branch, De Soto State Park, Fort Payne, 13 Sep 1962, *Demaree 46247* (NY). **Arkansas**, Ouachita Region, Garland Co., Elakely Creek, Buckville, 24 Oct 1968, *Demaree 59628* (SMU); Pike Co., Langley, 30 Oct 1975, *Demaree* *70465* (RSA). **Arkansas**, Pulaski Co., Des Moulins, 27 Sep 1931, *Demaree 8260* (NY). **Missouri**, St. Louis, Aug 1845, *Engelmann* *s.n.* (MO). ***Cuscuta coryli*** Engelm.: **CANADA, Manitoba.** District Provencher between Saint-Pierre and Otterburne, 27 Aug 1956, *Bernard 5669* (DAO); District Selkirk, Rivière Rouge, 23 Aug1960, *Boivin & Champagne 13869* (DAO, UNB). **U.S.A. West Virginia**, Ohio Co., on bank of Ohio River just below Eight Street, Wheeling, 16 Sep 1951, *Bartholomew 0-923* (NY). ***Cuscuta corymbosa*** Ruiz & Pav. var. ***grandiflora*** Engelm. **MEXICO. Baja California Sur**, Gulf of California, Isla Partita, 22 Apr 1921, *Johnston 3222* (GH). **Jalisco.** Sierra de Manantlán Occidental, Arroyo Lay Joyas, 19**°**35’15” 45°N, 104°15’30’ 45” W, 1 Jan 1984, *Iltis & Guzman 29077* (MICH). **State of Mexico**, Temascaltepec, Cumbre-Cruz, 15 Mar 1936, *Hinton et al. 8984* (GH). ***Cuscuta corymbosa*** Ruiz & Pav. var. ***stylosa*** (Choisy) Engelm.: **MEXICO. Hidalgo**, Mpio. San Salvador, km 135–137 on Laredo Hwy. between Actopan and Ixmiquilpan, 8 Oct 1943. *Gilly & Cany 5* (MICH). **Veracruz**, Dec 1915, *Purpus 7564* (GH). ***Cuscuta costaricensis*** Yunck.: **MEXICO. Chihuahua**, Las Gallinas, +/- 15 km west of Yepachi, 28^o^27’ N 108^o^31’W, 1500 m, 22 Oct 1984, *Levin 1440* (SD). **Durango**, El Saltito, Nombre de Dios, 13 Sep 1984, *Jienez & Acevedo 35* (MEXU); San José de Parrilla, +/- 4 km al W of 23^o^43’99”N 104^o^8’99”W, 25 Oct 1983, *González & Acevedo 2758* (MEXU). **Jalisco.** Isla Alacranes (Chapala), Maleza, 17 Sep 1964, *Cota 77* (MEXU). **Michoacan**, Abadiano, 19°59'45.18"N, 102°51'41.04"W, 18 Feb 2018, *Costea & García* *s.n.* (CIMI, WLU)*. ***Cuscuta cristata*** Engelm.: **ARGENTINA. Buenos Aires**, Baradero, Estancia “Los Alamos”, 27 Oct 2003, *Robles 1511* (MO). **Catamarca**, Rio Alumbrara, (no date), *Riggs 100* (F). **Córdoba**, Río Terrero, 10 Jan 1940, *Burkart 10399* (MO). Rio Yercero, entre Almafuerte y el Embalse del Rio III, 27 Feb1944, *Hunziker 4927* (S). ***Cuscuta cuspidata*** Engelm.: **U.S.A. Indiana**, Posey Co., along the Wabash River, 2 mi S of New Harmony, 24 Sep 1920, *Deam 33011*(NY). **Kansas**, Meade Co., 18 Sep 1944, *Horr E476* (BRIT, SMU). **Missouri**, St. Louis, 11 Aug 1891, *Eggert s.n.* (CAS). **New Mexico**, Clayton, 24 Sep 1907, *Evans s.n.* (NMC). ***Cuscuta denticulata*** Engelm.: **U.S.A. California**, Inyo Co., canyon leading into Saline Valley 36˚34’N, 117˚35’W, 16 Sep 1960, *Thomas 8904* (RSA); 1 mi E of Ballarat, 12 May 1947, *Munz 11731* (SD, UCR). Hwy 178, 3 mi E of Shoshone, 23 Jun 2013, *Stefanović SS-13-28* (WLU, TRTE). Riverside Co., Morongo Wash at San Bernardino Co. line, 11 Oct 1932, *Wolf 4282* (BRIT). San Diego Co., Borego Valley, 7 Apr 1940, *Howe 991* (SD). **Nevada**, Esmeralda Co., Intersection of Hws. 264/6, 25 Jun 2013, *Stefanović SS-13-39* (TRTE, WLU). ***Cuscuta desmouliniana*** Yunck.: **MEXICO. Baja California**, Chavez Ranch airstrip, ca. 5 mi NW of Mulegé, 22 Oct 1962, *Wiggins 18130* (MEXU). **Sonora**, Hills near Altar, 26 Aug 1884, *Pringle s.n.* (NY); 15 mi. S of La Paline, 2 Sep 1941, *Wiggins & Rollins 232* (GH); Hwy. W from Hermosillo to Bahía Kino, 29 Jan 1963, *Dunn et al. 14130* (NY); ca. 10 mi. due S of Desemboque, 22 Mar 1978, *Spellenberg 4943*(MEXU, NMC); Terrace above arroyo at mouth of Nacopuli Canyon, N of San Carlos, 28^o^01’N, 111^o^03’W, 26 Apr 1985, *Burgess 6949* (MEXU). ***Cuscuta draconella*** Costea & Stefanović: **U.S.A.** **Texas**, Brewster Co., Hwy 118, 47(A)-52(B) mi S of Alpine, 2016, *Stefanović SS-16-71*(TRTE, WLU). ***Cuscuta epilinum*** Weihe: **CANADA. Quebec**, 4 Aug 1880, *Pringle 204788* (CAS); Shefford, Saint-Alphonse, 30 Jul 1941, *Cartier s.n* (DAO, QFA); Saint-Alphonse, 30 Jul 1941, *Barabe 16914* (BRIT, SMU); Sainte-Hélène, Kamouraska, 15 Aug 1942, *Cayoutte s.n.* (QUE). ***Cuscuta epithymum*** L.: **AUSTRALIA. Victoria**, Rd. To Mt. Beauty, 19 Feb 1986, *Clarke 107955-212* (RSA). **CANADA. British Columbia**, Kootenay Lake, Balfour Bay, 16 Aug 1947, *Turner 5983* (ALTA). **MEXICO.** State of Mexico, Valley of Mexico, 27 Jun 1901, *Pringle 8514* (NMC). **SPAIN***.* **Andalucía**, Cabo de Gata, 36°49′11.96"N, 2°6’ 8.85” W, 24 Mar 2018, *Costea s.n.* (WLU)*. ***Cuscuta erosa*** Yunck.: **U.S.A. Arizona**, Pima Co., Baboquivari Canyon, *Kearney & Peebles 10422* (CAS, RSA); *Jones 28731* (CAS); Fresnal, 21 Aug 1932, *Peebles 8998* (QUE). **MEXICO. Sonora**, 15 mi SE of Magdalena on Rd to Cucurpe, 11 Sep 1934, *Wiggins 7123* (NY); Agua Prieta, ca. 7.5 km SW of Agua, 31˚15’34”N, 109˚36’34”W, 3 Oct 2004, *Van Devender et al. 2004-1199* (WLU). ***Cuscuta europaea*** L.: **BELGIUM. Prov de Luxembourg**, Chassepierre, 30 Sep 1975, *Duvigneaud & Lambinon 75B953* (QUE). **DENMARK. Dyrehaven**, Copenhagen, 6 Aug 1970, *Svedsen 329* (NY). **FINLAND. Kemiö**, Stenhol, 60˚05’N, 22˚45’E, 16 Aug 1978, *Alava et al. s.n.* (NY, RSA). **GERMANY. Alsfeld**, Kestrich, Sengesweg, 12 Aug 1972, *Hupke s.n.* (USAS). **Thüringen**, 20 Aug 1900, *Rudolph s.n* (RSA). **NETHERLANDS. Gelderland**, near Tiengeboden, 12 Sep 1959, *Hekking 635* (NY). ***Cuscuta foetida*** Hook. & Arn. var. ***foetida:*** **EQUADOR. Azuay**, Laguna Llaviuco (Surucuchu), W of Cuenca, 25 Feb 1993, *Harling & Ståhl 26675* (S). **Cañar**, Parroquia Bayas, Valley of Rio Tabacal, ca. 15 mi NE Azugues, 27 Sep 1944, *Prieto P-111* (S). **Chimborazo**, Rd from Sibambe to Alausí, 25 Aug 1943, *Solis 599* (F). ***Cuscuta gigantea*** Griff.: **INDIA, Jammu and Kashmir** [no locality, precise date], 1925, *Stewart 8249a* (NY). ***Cuscuta glabrior*** (Engelm.) Yunck.: **MEXICO, Coahuila**, Parras de la Fuente, Sierra de Parras, Rancho El Tunal, 21 Aug 1982, *Cowan 3644*(MEXU); ca. 35 miles SSW of Cuatro Cienegas, ca. 9.2 mi S of El Hundido, 26^o^30’N, 102^o^17’W, 29 Sep 1973, *Henrickson 13676c*(RSA); 27 mi SE of Saltillo, 16 Jul 1905, *Palmer 723*(GH, MO). ***Cuscuta globulosa*** Benth.: **CUBA. Oriente**, El Cobre, 6 Oct 1916, *Ekman 7839* (S). **PUERTO RICO. Culebra**, Playa Flamenco, at end of road along beach, 15 Jul 1989, *Axelrod 1154* (UPRRP). **Guayama**, Bo. Palmas, Rte 712, km 16.7, 1 km along farm track following ridge S of road, 21 Jan 1991, *Axelrod 1875* (UPRRP). **U.S.A. Texas**, Deaf Smith Co., 15 mi N & 15 mi W of Hereford, 23 Jul 1966, *Waller 962* (TEX, LL). ***Cuscuta glomerata*** Choisy: **U.S.A. Indiana**, Lake Co., 2 mi N of Hobart, 17 Sep 1930, *Deam 49686* (NY). **Kansas**, Riley Co., NW ¼ sec. 13, T11S, R7E, 10 Sep 1979, *Freeman 293* (NY). **Nebraska**, Minden, Sep 1996, *Haperman 21141* (NMS). ***Cuscuta goyaziana*** Yunck. **BRAZIL**. **Goiás**, Sierra Dourada, 30 Jul 1952, *Macedo 3771* (S). ***Cuscuta gracillima*** Engelm.: **MEXICO. Sinaloa**, Mazatlan, 23˚12’N, 106˚25’W, 20 Nov 1926, *Jones 22408* (UCR). **State of Mexico**, 3 km W of Ixtapan de Oro, 19˚15’N, 100˚16’W, 3 Dec 1983, *Solheim & Benz 1073* (NY); Temascaltepac, 4 Nov 1932, *Hinton 2497* (F). ***Cuscuta grandiflora*** Kunth: **ECUADOR. Carchi**, Tulcan Canton, Colonia Huaqueña, Loma El Corazón, 77˚42’W, 00˚35’N, 8 Jul 1992, *Tipax et al. 1663* (QCNE, MO); Ca. 2 Km along the Rd El Angel-Tulcán, 77˚55’W, 0˚38’N, 14 May 1973, *Holm-Nielsen et al. 5214* (AAU); Km 3 on Rd El Angel-Tulcan, 77˚55’W, 00˚39’N, 13 Aug 1985, *Laegoord 54907A* (QCNE). **PERU. San Sebastián**, Cusco, 25 Apr 1925, *Pennell 13613* (F). ***Cuscuta gronovii*** Willd. ex Schultes var. ***gronovii*: CANADA**. **Ontario**, Norfolk Co., Near Long Point Provincial Park, 42˚35’43.34”N, 80˚27’2.66”W, 19 Oct 2014, *Costea & Ho s.n.* (WLU); Wellington Co., Waterloo along the Grand River, Oct 2014, *Ho s.n.* (WLU); Glengarry Co., Kenyon Twp., 10 km NE of Maxville, 7 Sep 2001, *Brownell& Catling s.n*. (DAO). **Quebec*,*** Cté de Chauveau, St-Augin, 14 Aug 1975, *Pérusse 75-391* (QFA); Comté de Lotbinière, Sainte-Croix-de-Lotbinière, 46˚39’10”N, 71˚49’30”W, 24 Oct 1989, *Garneau & Roy 89-626-M* (QFA). Reserve faunique du lac Saint-Francis, 45°02’ N, 74°30’W, 1 Sep 1988, *Dignard & Masson 1101* (QUE); Cté. D’Argeuteuil, 10 Aug 1986, *Guertin 1334* (QFA). **U.S.A.**, **Florida**, Nassau Co., ca. 10 mi NE of Yule, *Wilbur 69645* (BRIT). **North Carolina**, Macon Co., 15 Sep 1910, *Anderson 1858* (SMU). **Ohio**, Hocking Co, Good Hope Twp., 1.25 mi SW of center of Rockbridge, 16 Sep 1979, *Bryant 1016* (QFA). ***Cuscuta harperi*** Small: **U.S.A. Alabama**, De Kalb Co., De Soto State Park, 13 Sep 1962, *Demaree 46295* (NY); Marion Co., North Fork Creek, S of Hackleburg on US 43, 7 Sep 1968, *Kral 32878* (SMU). ***Cuscuta huaghtii*** Yunck.: **EQUADOR, Guayas**, Salinas, La Puntilla, 6 Apr 1939, *Asplund 5618* (G, S). **Manabi**, Manta, 6 Apr 1955, *Asplund 15974* (UPS). ***Cuscuta howelliana*** P. Rubtzoff: **U.S.A. California**, Peter Ahart Ranch, Honcut, Valley Grassland, 7 Apr 1978. *Ahart 1804* (CHSC); Shasta Co., between Goose Valley and Burney Valley, ca. 3.5 mi. NNW from Burney, 8 Aug 1988, *Taylor 10026* (CAS). ***Cuscuta iguanella*** Costea & I. García: **MEXICO. Jalisco**, Wooded hills near Guadalajara, 2 Sep 1893, *Pringle 4529* (GH); near Guadalajara, 30 Sep 1903, *Rose & Painter 7473* (NY). ***Cuscuta incurvata*** Progel: **PARAGUAY. North Paraguay**, In regione cursus superioris fluminis Apa, Dec 1901, *Hassler 8178* (F); Zwischen Rio Apa und Aquidaban, 1908/1909, *Fiebrig 5083* (K). **Caaguazú.** 4 km al N de Yhú 6 Feb 2003, *López et al. 243* (CTES). ***Cuscuta indecora*** Choisy var. ***indecora***: **HONDURAS.** Malfredi Lagoon, 1 May 1933, *Schipp 1161* (S). **U.S.A. Arizona**, Gila Co., along rd. N of Winkelman, S of Globe, 16 Aug 1973, *Moldenke 27920* (AAU); Pinal Co., near Peppersauce Campground on N side of Santa Catalina Mountains, ca. 15 miles SE of Oracle, 30 Aug 1989, *Austin 7599* (RSA). **Nebraska**, Arthur Co., Arapaho Prairie, T18N R39W Sect 31, 32, 27 Jul 1977, *Vescio & Kruse 174* (NY). **Nevada**, Nye Co., U.S. Atomic Energy Commission test site and vicinity, Rt 52 near Rt 16 jnct., 26 Sep 1970, *Beatley s.n.* (RSA). **New Mexico**, Chaves Co., E side of Pecos River, E of Rosvell, 25 Jul 1973, *Spellenberg et al. 3427* (NY). **Utah**, Salt Lake City, 25 Aug 1879, *Jones s.n.* (RSA). ***Cuscuta jalapensis*** Schltdl.: **GUATEMALA. Totonicapán**, Region of Chui-quisís, above Totonicapán on Rd. to Desconsuelo, 23 Jan 1948, *Standley 84397*(NY). **MEXICO. Chiapas**, Amatenango del Valle, 26 Jul 1966, *Breedlove 14669*(MICH); 26 Jul 1966, *Breedlove 15669* (MICH); San Cristóbal de Las Casas, 15 Nov 1986, *Ton & Lopez 9826*(GH); Tenejapa, Paraje Shohleh, 12 Jan 1966, *Ton 603*(NY). **Hidalgo**, El Chico, vicinity of Peña del Cuervo, 5 km SE of El Chico, 20 Jul 1986, *Medina 3102*(MICH). **Puebla**, Near Huauchinango, 27 Mar 1945, *Sharp 45380*(NY). ***Cuscuta japonica*** Choisy var. ***japonica***: **CHINA. Guizhou**, Songtao Xian, vicinity of Lengjiaba, 5–9 Oct 1986, *Bartholomew et al. 2309* (RSA). **Shaanxi**, Foping, 15 Oct 1998, *Li 619* (MO). **JAPAN. Okayama**, Bizen, 7 Oct 1925, *Masamune s.n.* (NY). **Hondo**, 17 Oct 1952, *Hashimoto 853* (NY). **Honshu**, Kyoto, Anshu, Yamashina-ku, Kyoto-shi, 25 Oct 1998, *Tsugaru et al. 27202* (MO). ***Cuscuta legitima*** Costea & Stefanović: **MEXICO. Baja California**, 3.5 mi S of La Paz, ca. 24˚09’N, 100˚15’W, 3 Nov 1959, *Wiggins 15294* (MEXU). **Sonora**, Cerro La Antenna, 1 km N of Microondas, La Cabana, 27**°**27’45” N, 109°46’20” W, 19 Sep 1994, *Van Devender 94-603* (ARIZ). **U.S.A. Arizona**, Douglas, 11 Sep 1948, *Jones s.n.* (RSA). **New Mexico**, Maricopa Co., ca. 80 Km W of Phoenix, 18 Aug 1999, *Spellenberg & Zucker 12966* (NMC); Dona Ana Co., 15 Sep 1951, *Dunn 3850* (RSA). ***Cuscuta liliputana*** Costea & Stefanović: **U.S.A. New Mexico**, Sierra Co., 3 mi S of Hillsboro, 9 Sep 1904, *Metcalfe 1290* (SD). **Texas**, Hildalgo Co.: 4.5 mi S of San Juan, 6 Feb 1969, *Correll 36759* (UC). ***C. leptantha*** Engelm.: **MEXICO. Baja California**, 10 mi inland from Bahia de Los Angeles, near Agua Armaga, ca. 29˚00’N, 113˚45’W, 22 May 1960, *Lindsay 2928* (SD); Los Animas Bay, 8 May 1928. *Johnston 3484* (GH); Between Santonrio and Puerto de Bahia de los Muertos, 4 May 1931, *Wiggins 5625* (GH); near Club Aereo airport, Mulegé, 9 Apr 1963, *Wiggins & Wiggins 18219* (K); Bahia de Los Angeles, SW shore, 14 Mar 1992, *Fritsch & Fritsch 1337* (RSA). **Cuscuta lupuliformis** Krock. **GERMANY**. **Rheinland-Pfalz**, Biewer, 14 Sep 1977, *Duvigneaud 77 A884* (MEXU). **HUNGARY**. Borsod-Abaúj-Zemplén Co., Tiszaújváros, 47.95° N, 21.07° E, 2017,  *Lovas-Kiss s.n.* (WLU). ***Cuscuta macrocephala*** W. Schaffn ex. Yunck.: **MEXICO. Baja California**, 7.1 mi S of Caduano, between Sauta Anita and La Palma, 18 Jan 1959, *Wiggins 14726* (GH, OSU); La Paz, 20 Jan-5 Feb 1890, *Palmer 141* (GH); Along rd to El Valle Perdido, 4 mi. E of La Paz Todos Santos rd, about 23^o^43’N, 110^o^10’W, 6 Nov 1959, *Wiggins 15323*(K). **Sinaloa**, Cosalá, 24°24'16"N, 106°41'26"W, 4 Jan 2006, *Van Devender 2006-15*(WLU). ***Cuscuta membranacea*** Yunck.: **ARGENTINA. Catamarca Prov.**, La Ramadita, 17 Feb 1944, *Hunziker 4823* (S). **La Rioja Prov.**, La Rioja, 9 Mar 1944, *Hunkziker 4695* (S); Los Sauces, Mar 1944, *Hunziker 4833* (S). **Mendoza**, San Rafael, Valle del Rio Atuel, 26 Jan 1963, *Boelcke et al. 10243* (CTES). ***Cuscuta microstyla*** Engelm.: **ARGENTINA.** *Boelcke et al. 10243* (CTES). ***Cuscuta mitriformis*** Engelm.: **MEXICO. Chihuahua**, Sierra Madre, 2 Oct 1887, *Pringle 1342* (MO). **Coahuila**, ca. 22 mi WNW of Cuatro Cienegas, Canyon de la Hacienda, Sierra de la Madera, ca. 27˚04’N, 102˚25’W, 28 Sep 1973, *Henrickson 13638* (RSA). **Michoacán**, Mountains, near Lake Chapala, 19 Nov 1892, *Pringle 4330* (S); ca. 18 mi S of Pátzcuaro, 20-25 Nov 1961, *King & Soderstorm 5214* (MICH, NY). **Nuevo Leon**, Mpio. Galeana, Galeana, Hacienda Pablillo, 1 Aug 1936, *Taylor 38* (GH). ***Cuscuta monogyna*** Vahl: **GREECE.** Distr. Istiea, Lihadha, 29 Jul 1973, *Greuter 11459* (OSU). **ISRAEL**. Kursi, 32°49′34.9′′N, 35°38′59.6′′E, 2012, *Wizen s.n*., UTM‐1348 (WLU, TRTE). **UZBEKISTAN.** 19 Jul 1914. *Knowing 74* (NY). ***Cuscuta montana*** Costea and Stefanović: **MEXICO. Durango**, Mpio. El Salto, along Mexico Hwy 40, 0.6 mi W of Los Angeles and 3.6 mi E of Revolcaderos, 27 Sep 1973, *Reveal & Atwood 3616* (NY); Along Hwy 40, 10.6 mi El Palmito, 30 Dec 1962, *Breedlove 4261*(MICH). ***Cuscuta nevadensis*** I.M Johnst.: **U.S.A., California**, Inyo Co., lower end of Westhaard Grade, near Big Pine, 19 Jul 1969; San Bernardino Co., Ridge top NW of Indian Pass, 1 Jun 1983, *Peterson 888* (NY). **Nevada**, Amargosa, 19 Jun 1969, *Beatley 9055* (RSA); Steward Valley, 12 Apr 1970, *Beatley 9954* (RSA). ***Cuscuta nitida*** Meyer.: **SOUTH AFRICA. West Cape**, Clock Peaks, near Swellendam, 11 Dec 1973, *Carlquist 5082* (RSA). **Cape Peninsula**, Smitswinkel hillside, 11 Jan 1944, *Compton 15508* (NBG). ***Cuscuta obtusiflora* Kunth** var. ***glandulosa* Engelm.**: **GUATEMALA.** **Jutiapa**, Lago Retana, between Ovejero and Progreso, 26 Nov 1939, *Steyermark 31988* (F). **MEXICO. Tamaulipas**, Hacienda Santa En Gracia, 24 Jul 1939, *Chase 7563* (NY). **U.S.A. Arkansas**, Ashley Co.: Lone Prairie, 15 Sep 1940, *Demaree 21543* (MO). **Texas**, Dallas Co., Dallas, [no date], *Reverchon 2878* (MO); Bachmans Dam, 24 Aug 1944, *Lundell 11626* (SD); Rio Grande, 1848, *Wright s.n.* (MO); Bastrop Co., Sep 1937, *Tharp s.n.* (MO). ***Cuscuta occidentalis*** Millsp.: **U.S.A. California**, Los Angeles Co., San Clemente Island, SW of the new landing field, 17 May 1991, *Ross et al. 5087* (RSA); Marin Co., Mt. Tamalpais, 8 May 1922; Siskiyou Co., Siskiyou Mts., Lily Pad Lake, 21 Aug 1958, *Wheeler 7417* (RSA). ***Cuscuta odontolepis*** Engelm.: **MEXICO. CHIHUAHUA**, Nobogame, NW of Yepachic, 23 Oct 1986, *Leferriere 318* (MEXU). **Sonora** [unknown locality], 1851-1852, *Wright 1624* (K); Near a deserted Rancho on rocky hill sides, 15 Sep 1851, *Wright 529* (MO). ***Cuscuta odorata* Ruiz & Pavon** var. ***odorata***: **ECUADOR. Chimborazo**, Cañon of the río Chanchan near Huigra, 7–14 May 1945, *Camp 3027*(S). **PERU. Dept. Lima**, San Mateo, 28 May 1940, *Asplund 11177*(S); Lima, along Rio Chillón, above Obrajillo, 13–23 Jun 1925, *Pennell 14382*(S). ***Cuscta pacifica*** Costea & M.A.R Wright: **CANADA. British Columbia**, Vancouver Island, vicinity of Victoria, 24 Jul 1983, *Macoun 695* (K). **U.S.A. California**, Humboldt Co., Humboldt Bay near Table Bluff, 28 Aug 1941, *Harris & Harris 1175* (NY); Marin Co., 5 Nov 1939, *Howell 15386* (UC). **Oregon**, Lane Co., Marshy tide land at Florence, 28 Oct 1930, *Fletcher s.n.* (OSC). ***Cuscuta paitana*** Yunck.: **ECUADOR.** Isla Puná, Path from Campo Alegre to midway Rio Hondo, 7 Jul 1997, *Madsen 63790* (AAU). **PERU. Dept. Piura**, Paita, La Brea, 16–17 Mar 1927, *Weberbauer 7762* (F); Pariñas Valley about 6 mi inland, 26 Dec 1928, *Haught 100* (F). ***Cuscuta parodiana*** Yunck.: **ARGENTINA. Prov. La Salta**, La Caldera, Yacones, 29 Apr 1990, *Novara & Bruno 9821* (S); Rio San Alejo, 1 May 1987, *Novara 6656* (S). **Prov.** **Jujuy**, Ledesma, Rd from Faile Pintado to El Aibal, 13 Nov 1992, *Kiesling 8236* (MO); 26 Feb 1901, *Kurtz 11792* (S). ***Cuscta partita***Choisy: **BRAZIL. Maranhão**, Lorêto, “Ilha de Balsas region”, between the Balsas and Parnaíba Rivers, ca. 35 km S of Lorêto, 7^o^23’S, 45^o^4’W, 3 Apr 1962, *Eiten & Eiten 3961*(US); 22 Feb 1970, *Eiten & Eiten 10752* (US). **Piaui**, Corrente, BR-135, 10^o^27’S, 45^o^9’W, 3 Apr 1983, *Krapovickas et al. 38723*(CTES).**COLOMBIA.**Rincon Hondo, Magdalena Valley, 5 Aug 1924, *Allen 267*(F, MO). ***Cuscuta pentagona*** Engelm.: **U.S.A. Florida**, Levy Co., Cedar Key, 10 May 1958, *Godfrey 5650* (NY). **Indiana**, Cass Co., ca.1.5 mi NW of Lake Cicott, 1 Oct 1940, *Deam 60219* (IND); Starke Co., 2.5 mi SE of North Judson, 18 Jul 1920, *Deam 49139* (IND). **Massachusetts**, Onset, 27 Aug 1901, *Edmondson 2777* (NY). **Michigan**, Kalamazoo Co., Fort Custer, 12 Aug 1945, *Hanes 4541* (NY). **Texas**, Hunt Co., 7.4 mi E of Greenvile, 8 Jun 1953, *Shinners 15030* (TEX/LL). ***Cuscuta planiflora*** Ten.: **PALESTINE.** Near village of Kesan, near Tekoa, 31˚35’N, 35˚15’E, 22 May 1987, *Musselman 10461* (RSA). [Unknown locality, undated], *Priva 82* (S). ***Cuscuta platyloba*** Progel: **ARGENTINA. Misiones**, San Ignacio, Balneario Teyú Cuaré, 27^o^16’S 55^o^35’W, 23 Nov 1995, *Guaglianone et al. 3025* (K). ***Cuscuta polyanthemos*** W. Schaffn. ex Yunck.: **MEXICO. Sinaloa**, 22.6 mi S of Culiacan, *Wiggins 13153* (SD). **Sonora**, Monctezuma, 29˚39’44” N, 109˚37’13” W, 14 Sep 2006, *Van Devender 2006-809* (WLU); 31 mi NW Quiriego, 6 Mar 1933, *Wiggins 6457* (US). ***Cuscuta polygonorum*** Engelm.: **U.S.A.** **Arkansas**, St. Francis Co.: 5 mi S of Forrest City, 19 Sep 1959, *McDaniel 1419* (NY). **Indiana**, Grant Co., ca. 5 mi NE of Fairmont, 4 Sep 1914, *Deam 15269* (IND); ca. 1/4 mi N of Fredonia, 2 Oct 1924, *Deam 48024* (IND). ***Cuscuta purpurata*** Phil.: **CHILE**. **Region III**, Atacama, Parque Nacional Pan de Azúcar, 5 Dec 1987, *Dillon & Teiller 5104* (MO); Desert of Atacama Sep -Oct 1890, *Thos. Morong 1143* (US). **Antofagasta**, Quebrado de Taltal*,* vicinity of Taltal, 25 Nov 1925, *Johnston 5082* (US). ***Cuscuta purpusii*** Yunck.: **MEXICO.** Mpio. General Zaragoza, Cerro el Viejo, Zaragoza, 23 Sep 1993, *Hinton et al. 23503* (ARIZ). **Nuevo Leon**, *Henrickson 6608* (RSA). **San Luis Potosí**, Guadalcázar, Cerro El Calvario, 22˚36’0” N, 100˚23’0” W, 31 Jul 2000, *Torres Colín 15864* (MEXU). ***Cuscuta reflexa*** Roxb.: **INDIA.** Uttar Pradesh, Allahabad, 23 Jan 1923, *Dudgeon s.n.* (NY); 25 Jan 1923, *Dudgeon s.n* (NY); ***Cuscuta rostrata*** Shuttlew. ex Engelm. & A. Gray: **U.S.A. North Carolina**, Watauga Co., along South Fork of New River, 0.8 mi. S of Todd on Rt. 1347, 20 Sep 1968, *Leonard* et al. *2053* (NY); Mitchell Co., 0.6 mi W SW of Hughes Gap on rd. to Buladean, 25 Sep 1958, *Ahles & Duke* 49884 (NY). **Virginia** [No location], 28 Aug 1939, *Alexander s.n.* (NY). ***Cuscuta rugosiceps*** Yunck.: **MEXICO, Oaxaca**, Sierra Madre del Sur, 20 Jun 1962, *Webster 11561* (GH). Sierra de San Felipe, 6 Oct 1894, *Pringle 4967* (NY). **Queretaro**, Jalpan, 21˚28’11” N, 99˚09’02” W, 3 Sep 2005, *Pérez-Calix 4603* (IEB, WLU). ***Cuscuta salina*** Engelm. **U.S.A., California**, Madera Co.: 4 mi SW of Chowchilla, 1 Oct 1936, *Hoover 1610* (UC); Glen Co., Sacramento National Wildlife Refuge, 5 Aug 1993, *Oswald 5777* (CHSC); Fresco Co., 1 mi. W of Kerman Junction, on California Hwy. 180, 29 Jul 1941, *Bacigalupi* *et al*. 2667 (UC). **Nevada**, Caliente, 27 Aug 1912, *Jones s.n.* (RSA). ***Cuscuta sandwichiana*** Choisy: **U.S.A. Hawaii**, Mahana, Lanai, 20 Aug 1957, *Degener 24212* (RSA); Molokai, E. Fork of Kawela Gulch, 5 May 1928, *Degener & Wiebke 3261* (NY); Oahu, Kaena, 11 Mar 1947, *Cowan 730* (NY). ***Cuscuta sidarum*** Liebm.: **MEXICO. Michoacán**, Mpio. de Arteaga, along the road to Infiernillo, 6.2 km SSE of the junction with MEX 37, 18˚27’55” N, 101˚58’40” W, 20 Nov 2003, *Steinmann 3883* (IEB). **Yucatán**, Chocholá, 25 km al SW of Mérida, 20˚44’30”N, 89˚47’20”W, 7 Nov 2001, *Carnevali et al. 6425* (CICY); “S. Jago Estate” [unknown locality], Nov 1841, *Liebmann 12359* (S). ***Cuscuta squamata*** Engelm.: **U.S.A. New Mexico**, Doña Ana Co., White Sands Missile Range, 3 mi E of Main Post, East Dry Lake Playa [no date], *Anderson & Brice 8057* (NMC); Mesa W of Organ Mountains, [no date], *Wooton s.n.* (NMC). **TEXAS**, Garza Co., 11.1 mi NW of Post, *Hutchins 643* (SMU); Culberson Co., between Texline and Orla, 7 Oct 1951, *Warnock 10275* (SMU); El Paso, 10 Sep 1883, *Jones 4170* (RSA). ***Cuscuta stenolepis*** Englem.: **ECUADOR. Prov. Imbabura**, Ibarra, 15 Dec 1956, *Weydahl 407* (S). **Prov. Pinchicha**, Rd. Mitad del Mundo-San José de Minas, 26 Apr 1980, *Jeramillo & Carvajal 2307* (AAU); Tabacundo, entrada a Cochasqui, 18 Jan 1992, *Palacios & Tipaz 9636* (MO). ***Cuscuta strobilacea*** Liebm. var. ***pringlei*** (Yunck.) Costea & I. García: **MEXICO, Guadalajara**, La Barranca, 25 Nov 1930, *Jones 97347* (MICH); La Barranca, 23 Nov 1930, *Jones s.n.* (RSA). ***Cuscuta suaveolens*** Seringe: **U.S.A. California**, Humboldt Co., Myers Ranch, in alfalfa field, South Fork of Eel River 8 miles above the mouth, 29 Sep 1918, *Tracy 5113* (JEPS, UC1); Kern Co., Rosedale, 30 Sep 1894, *Abrams 458* (RSA). ***Cuscuta subinclusa*** Durand & Hilg.: **U.S.A. California**, Kern Co., Kernville, 25 Sep 1970, *Howell 47416* (NY); Riverside Co., San Gorgonio, San Gorgonio Pass, 1 Dec 1933, *Wheeler 2284* (RSA); San Diego Co., 4 mi W of Hwy 94 on road to Otay Reservoir, N base of San Ysidro Mts., 20 Aug 1952, *Munz & Balls 17942* (NY); San Luis Obispo Co., Rinconada District, below Santa Margarita and Pégo, 15 Sep 1946, *Hoover 6401* (RSA). ***Cuscuta suksdorfii*** Yunck.: **U.S.A. California**, Plumas Co.: Lost Lake, ca. 2 mi NNW of Humbug Summit. 22 Aug 1989, *Oswald & Ahart 3949* (CHSC); Mariposa Co., Yosemite National Park, 37.82630N, 119.49910W, 20 Jul 2004, *Colwell* *& Sanders AC 04-159* (UC); Siskiyou Co., S side of Preston Peak, Rattlesnake Meadow, 25 Aug 1963, *Wheeler 8269* (RSA). ***Cuscuta tasmanica*** Engelm.: **AUSTRALIA, New South Wales**, W shore of Lake Bathurst, ca. 2 km NE of Tarago, 35˚03’S, 149˚39’E, 28 Dec 1992, *Lepschi 908* (CANB). **Victoria**, Moyne-North-West, between Lake Corangamite and Lake Martin, W of Berrybank-Cundare Road, 29 Jan 1991, *Walsh 3045* (MEL). ***Cuscuta timida*** Costea & Stefanović: **MEXICO.** **Hildago**, Km 134.5 near Yolotepec, 8 Jul 1943, *Lundell 12538* (MICH). **Veracruz**, Mpio. Maltrata, Maltrata, Jan 1883, *Kerber 248* (UPS). ***Cuscuta tinctoria*** var. ***floribunda*** (Kunth) Costea: **MEXICO. Estado de Mexico**, Mpio. Temascaltepec, E of La Peña, on Rd toTemascaltepec ca. 13.5 km of SE Valle de Bravo, 27 Dec 2012, *Garcia Ruiz et al. 8588* (CIMI). ***Cuscuta tuberculata*** Brandegee: **MEXICO. Baja California Sur**, N slopes of Varro Gabilán, S of Portezuelo de Gabilán, 25˚50¾’N 111˚25’W, 2 Oct 1965, *Carter 5080* (MICH); 12 mi NE Villa Insurgentes, 7 Sep 1983, *Donahue 73168* (RSA). **Sonora**, Bavispe, 19 Oct 1980, *Lumholtz 179* (GH). ***Cuscuta umbrosa*** Beyr. ex. Hook.: **CANADA, Manitoba**, Otterbourne, 4 Aug 1954, *Bernard 54/349* (QFA); District de Saint-Boniface, Riviere Rouge, 21 Aug 1960, *Boivin 13852* (DAO); 20 Aug 1953, *Lore & Lore 6199* (DAO); Sans Souci, 29 Jul 1956, *Bernard 56/5473* (QFA). **U.S.A. New Mexico**, Lincoln Co., White Mts., Little Creek, 19 Aug 1909, *Turner 271* (NMC). ***Cuscuta umbellata*** Kunth: **GUIANA.** Georgetown, 29 Oct 1919, *Hitchcock 16564* (S). **MEXICO. Oaxaca**, Mpio. San Juan Bautista Cuicatlán: 10.2 Km al NE of Chilar, 17°47′21”N, 96°59′31”W′, 1 Oct 2002, *Soto 24009* (MEXU); **Puebla**, near Tehuacan, 24 Dec 1895, *Pringle 6297* (S). ***Cuscuta vandevenderi*** Costea & Stefanović: **MEXICO. Sonora**, Sierra Tecurahui, 26–28 October 1961, *Gentry et al. 19423* (US); El Guayabo Crossing of Rio Cuchujaqui, 14 km ESE of Alamos, 21 Nov 1993, *Steinmann et al. 93-349*(ASU). ***Cuscuta veatchii*** Brandegee: **MEXICO. Baja California Norte**, Los Angeles Bay, 6 May 1921. *Johnston 3430* (GH); Close to Bahía de Los Ángeles, 28°58′47.1″ N, 113°43′13.0″ W, 30 April 2014, *Costea s.n.* (WLU); Cataviña Sur, ca. 60 km S of Cataviña, 30 April 2014, *Costea s.n.* (WLU). ***Cuscuta victoriana*** Yunck.: **AUSTRALIA. Canberra**, 6 mi NW of Mt. Swan Stations N.T, 11 Mar 1953, *Perry 3329* (CANB). **South Australia.** Region 2, Lake Eyre, between Hough’s Farm and Chapman’s Creek Tank, Dulkaninna Station, 29˚04’23” S 138˚37’28”E, 9 Apr 1997, *Smyth 261* (CANB). ***Cuscuta volcanica*** Costea & I. García: **MEXICO. Jalisco**, NE slopes of the Nevado de Colima, below Canoa de Leoncito, 10 Oct 1952, *McVaugh 13419* (MICH); Mpio. de Zapotitlán, 10 km on the exit to Nevado de Colima from Fresnito, 6 Apr 1988, *Mendoza et al. 3817* (MEXU). ***Cuscuta warneri*** Yunck.: **U.S.A. Utah**, Millard Co., vicinity of Flowell, 15 mi W of Fillmore, 10 Sep 1957, *Warner s.n.* (NY). ***Cuscuta woodsonii*** Yunck.: **GUATEMALA**. **Dept. Huehuetenango**, Santa Eulalia, 19 Nov 1999, *Véliz et al. 99.7506* (MO). **PANAMA.** **Chiriquí**, vicinity of Casita Alta, Volcano Chiriquí, June 28–July 2, 1938, *Woodson et al. 950* (NY). ***Cuscuta xanthochortos*** Mart. ex Engelm. ***var. carinata*** (Yunck.) Yunck.: **PARAGUAY. Cordillera**, Cerrado forest, 25^o^07’S 57^o^19’W, 1 Jun 1993, *Zardini & Guerrero 35961* (MO, WLU). ***Cuscuta yucatana*** Yunck.: **MEXICO. Chiapas**, Municipo of Tenejapa, Paraje of Banabil, 10 Oct 1965, *Breedlove & Raven 12912* (F); 7 Nov 1971, *Breedlove & Smith 22017* (MEXU). **Yucatán**. Valladolid, 2 Jul 1932, *Steere 1695* (NY).
