# Supplementary material for: Cuscuta seeds: Diversity and evolution, value for systematics/identification and exploration of allometric relationships
Source: PLoS One. 2020 Jun 12;15(6):e0234627. doi: 10.1371/journal.pone.0234627 (PMC7292398; doi:10.1371/journal.pone.0234627)
Supplement: Attachment — Submitted filename: Cuscuta_seeds_Letter_ReviewerARGS.docx [file pone.0234627.s010.docx]

Dear Editor,

The submitted manuscript seems to me a very important contribution to the knowledge of seed anatomy, morphology, and evolution and development in the genus *Cuscuta*, which is not only of great economic importance, but also very relevant for laying a foundation for further understanding the evolution and dispersal mechanisms of this parasitic plant, with impact on host-parasite interaction studies (ecology and evolution). It is a very comprehensive work, with a great quantity of data that must have required years of study, and analysed with appropriate methodological strategies, to the best of my knowledge. It will also be a good model for similar studies in the family Convolvulaceae, which are very much necessary.

Therefore, I am very supportive of the publication of these research results, for even with sampling limitations, it will leverage an array of other studies in the fields of plant systematics, ecology and evolution in this, and other, groups of plants.

My most significant recommendations go towards the actual presentation of the results, i.e. how the manuscript is written and structured. The importance of making these data and analyses available is undeniable, but I would strongly recommend to present it in a more effective way.

I have felt that the research hypothesis and how the applied methodology will address them are not laid out clearly enough, and in my view the contents are mixed up both in the Introduction, Methodology and Discussion. A more structured organization of the contents would allow summarizing some parts thar are overlapping between sections, and expanding on some discussions which would be interesting to extend.

As I understand it, this study is decomposed in the following components:

1) What is the variation? Documenting the variation in seed morphology and anatomy and coding this variation for further analysis; also, explicitly laying out the taxonomic and biogeographic scope of the study (if it is not the same for each section, I do not see a problem, but it needs to be explicitly defined from the outset).
2) How has this variation evolved in the group? Testing character evolutionary hypothesis in a molecular phylogenetic framework
3) Can this variation contribute to the systematics of the group? Given the character evolution, can synapomorphies be found for particular clades, and are they useful or not for diagnosing taxa of any particular rank?
4) Character correlations – do certain traits correlate to each other, and possibly why?
5) Additional information about seed development

The clear structure of contents would allow greater transparency and clarity in the communication of the hypotheses, and interpretation of the results. For instance, I understand that the characters explored did not contribute in a meaningful way to the systematics of the group. However, this is a result in itself, and further corroborates the already documented homoplasy present in so many characters in family Convolvulaceae. This needs to be openly addressed and discussed, as the other research questions.

My suggestion for a re-structuring of the manuscript is to

- offer the objectives in the form of research questions or scientific problems (in the way they are presented, they seem more like a narrative of the methodology)
- Present the results and discussion as answers to these research questions, therefore following a particular order of contents within each section, e.g. documentation of the variation, evolutionary analyses, value of characters for systematics and correlations between characters

For further details, I have added comments throughout the manuscript draft, but several of those will relate to this general organization of contents that I have laid out, and which I think hinders the value of this publication as is.

In summary, I recommend the manuscript to be accepted with major revisions, and I would be happy to revise an improved version.

Kind regards,

Ana Rita Simões.
