# Supplementary material for: Cuscuta seeds: Diversity and evolution, value for systematics/identification and exploration of allometric relationships
Source: PLoS One. 2020 Jun 12;15(6):e0234627. doi: 10.1371/journal.pone.0234627 (PMC7292398; doi:10.1371/journal.pone.0234627)
Supplement: Attachment — Submitted filename: Response to reviewers 2 Cuscuta Seeds PLOSone_ARGS(1).docx [file pone.0234627.s011.docx]

***Cuscuta* Seeds: Diversity and Evolution, Value for Systematics/Identification and Exploration of some Allometric Relationships**

Magdalena Olszewski^1^, Meghan Dilliott^1^, Ignacio García-Ruiz^2^, Behrang Bendarvandi^1^, Mihai Costea^1, *^

^1^ Department of Biology, Wilfrid Laurier University, 75 University Avenue W, Waterloo, Ontario N2L 3C5, Canada

^2^ Instituto Politécnico Nacional (CIIDIR-IPN Michoacán), Justo Sierra 28, Jiquilpan, Michoacán, México, C.P. 59510

*^*^ Corresponding author.* Tel.: 519-884-1970/3407

*E-mail address:* [*mcostea@wlu.ca*](about:blank) (M. Costea)

**Abstract**

*Cuscuta* (dodders) is a group of parasitic plants with tremendous economic and ecological significance. Their seeds, often described as “simple” or “unspecialized”, are important because, during this stage, they are dispersed or persistent in a seed bank. Previous studies of seed morphology and/or anatomy were conducted on relatively few species. We expanded research to 101 species; reconstructed ancestral character states; investigated correlations among seed characters and explored allometric relationships with breeding systems, the size of geographical distribution of species in North America, as well as the survival of seedlings. Seed morphological and anatomical characters permit the separation of subgenera, but not of sections. Identification of *Cuscuta* species using seed characteristics is difficult but not impossible if their geographical origin is known. Seeds of subg. *Monogynella* species, exhibit the likely ancestral epidermis type consisting of elongated and interlocked cells, which are morphologically invariant, uninfluenced by dryness/wetness. Subgenera *Cuscuta*, *Pachystigma* and *Grammica* have evolved a seed epidermis with isodiametric cells that can alternate their morphology between two states: pitted when seeds are dry, and papillose after seed imbibition. A seed coat with double palisade architecture has also apparently evolved in subgenera *Cuscuta*, *Pachystigma* and *Grammica*, but several species in two clades of the latter subgenus reverted to a single palisade layer. The same latter species also evolved a peculiar, globose embryo, likely having a storage role, in contrast to the ancestral filiform and coiled embryo present throughout the remainder of the genus. Autogamous species had, on average, the highest number of seeds per capsule, whereas fully xenogamous taxa had the lowest. No relationship was revealed between the size of the seeds and the size of their geographical distribution in North America, but seedlings of species with larger seeds survived significantly longer than seedlings which resulted from smaller seeds. Diversity and evolution of seed traits was discussed in relationship with their putative roles in germination and dispersal.

**Introduction**

*Cuscuta* (Convolvulaceae; dodders) is a genus of about 200 species of obligate parasitic plants with sub-cosmopolitan distribution (reviewed by [1]). Dodders are keystone species in their natural ecosystems impacting the diversity, structure and dynamics of plant communities [e.g., 2]. *Cuscuta* is one of the most economically detrimental groups of parasitic plants worldwide as infestation by some of its species can result in major yield losses in numerous crops [3–7].

Seeds are important because it is during this ontogenetic stage that dodders are either dispersed or persistent in a seed bank [6–8]. The worldwide anthropogenic dispersal of *Cuscuta* seeds, through contaminated commercial seed shipments and herbal products, has been well documented [e.g., 4, 6, 9]. The seeds of *Cuscuta* lack apparent morphological adaptations for a particular dispersal syndrome and have been considered “simple” or “unspecialized” [e.g., 10–12]. However, dodder seeds have been recently reported to be long-distance dispersed via bird endozoochory [13, 14], or water, in the species with indehiscent fruits [15].

In an effort to prevent and mitigate the threat of *Cuscuta* as invasive plants and agricultural weeds, quarantine legislation has been enacted worldwide [e.g., 6, 16], however, enforcing such legislation internationally is predicated on the ability to identify *Cuscuta* seeds. Morphological identification has prevailed in many phytosanitary labs worldwide because it is more expedient than the molecular approaches. Unfortunately, the taxonomy of *Cuscuta* species has historically relied on flowers and, to a less extent, on fruits [1, 15, 17, 18]. To date, the seeds of only 22 *Cuscuta* species have been studied; usually a few species at a time and often either morphologically or anatomically (summarized in Table S1; e.g., 9, 12, 19–24). Therefore, an overarching study of *Cuscuta* seeds with a broader taxonomic sampling is necessary to unify previous results, as well as to provide a comprehensive source of data for the comparison of seed characters with potential to be taxonomically informative at species level. Surveying the morphological and anatomical diversity of seeds in a phylogenetic framework (e.g., tracing character evolution) would also be important for the systematics of *Cuscuta* because of the scarcity of available morphological characters that bear a phylogenetic signal in this genus [1, 10, 25].

The dataset of seed traits resulting from this study can also be used to explore possible allometric or functional relationships of seeds in *Cuscuta*; for example, the average number of seeds produced per fruit and the breeding systems; the size of seeds versus the geographical distribution range of species, as well as seed size and the survival of seedlings. Broad-scale comparative studies of *Cuscuta* pollen/ovule ratios indicated that dodders possess a wide range a mixed mating systems, which ranged from functionally cleistogamous (and thus selfing) to obligate xenogamous [26, 27]. While the number of ovules per ovary is always four, pollen production by each flower varies over three orders of magnitude [27]. This allows testing the possible relationship between pollen ovule/ratios and the average number of seeds produced per capsule [e.g., 28–30]. Seed size has been related, in other angiosperms, to dispersal [e.g., 31–33) and seedling survival [34, 35]. This latter aspect is particularly important for *Cuscuta* population dynamics because, during this stage, they rely entirely on the nutritive reserves stored in the endosperm, although their seedlings are capable of uptaking water, and even form short-term associations with mycorrhizal fungi [36].. If seedlings cannot locate and attach to a compatible host within the short window of time provided by the seed reserves, they will die [4, 6]. Anecdotal *Cuscuta* seedling survival times reported in the literature (i.e., which did not result from a study) vary from eight days (*C. campestris*; [3]) to seven weeks (*C. gronovii*; [37, 38]), but to date there is no study comparing the seedling survival of several species of *Cuscuta*.

Thus, the objectives of this study are: (a) Survey the morphological and anatomical diversity of *Cuscuta* seeds and reconstruct ancestral character states; investigate correlations among continuous characters; discuss the usefulness of seed characters for species identification and their significance to the taxonomy and systematics of the genus; (b) Examine a putative relationship between the number of seeds per capsules and breeding systems in *Cuscuta*; (c) Establish if there is a relationship between seed size and the distribution range of species in North America; (d) Experimentally determine the effect of seed size on the seedling survival in three *Cuscuta* species.

**Materials and methods**

Taxonomic Sampling

The morphology and anatomy of seeds was examined in 101 *Cuscuta* species (Appendix S1). All the seeds were obtained from herbarium specimens and two to six specimens were examined per taxon (Appendix S1).

**Seed morphology and anatomy**

Ten seeds per herbarium specimen were used for external morphology using Scanning Electron microscopy (SEM). Seeds were rehydrated in a 50% ethanol solution brought to boiling point and preliminarily examined/imaged with a Nikon SMZ1500 stereomicroscope. Seeds were dehydrated through an ethanol series (50%, 70%, 85%, 95%, and 100 %; each step 1h) and then dried with a Tousimis Autosamdri-931 critical point dryer. Using SEM has been proven to be a valid approach to study not only the external morphology of seeds, but also the seed coat anatomy in *Cuscuta* [13, 14]. For anatomy, 5–10 seeds were sectioned longitudinally by hand with a razor blade along the hilum area. Longitudinal sections were dehydrated as for the SEM processing. Critically-point dried, entire and longitudinally sectioned, seeds were sputter-coated with 30 nm of gold using an Emitech K550 (Emitech, Ltd. Ashfort, UK). Imaging was completed using a Hitachi SU-510 variable pressure scanning electron microscope (SEM) at three kV. Character scoring and measurements were done using Quartz PCI version 5.1 (Quartz Imaging Corp.).

In addition, five seeds of 27 species (Subg. *Grammica: C. californica, C. campestris, C. argentiniana, C. cephalanthi, C. acutiloba, C. chapalana, C. chilensis, C. compacta, C. corymbosa, C. cristata, C. cuspidata, C. denticulata, C. desmouliniana, C. erosa, C. foetida, C. grandiflora, C. iguanella, C. indecora, C. mitriformis,* *C. veatchii*; subg. *Cuscuta*: *C. epilinum, C. epithymum, C. europea*; subg. *Monogynella*: *C. exaltata*, *C. gigantea, C. monogyna,* and *C. reflexa*) were also cross-sectioned by hand and processed for optical microscopy. These latter sections were stained with 0.05% Toluidine Blue O (TBO), a polymorphic stain (pH4.4; [39]); Sudan IV for lipids [40], and potassium iodide for starch (I^2^KI; [39]).

Seed water gap was studied in seven species from three of the four subgenera (*Monogynella*: *C. monogyna*, *C. lupuliformis*; *Cuscuta*: *C. epithymum; Grammica*: *C. gronovii, C. sandwichiana, C. tasmanica, C. veatchii, C. volcanica*) , with 20 seeds per species processed. . Physical dormancy was removed from 14 of 20 seeds using the rehydration protocol developed by Jayasuiraya et al. [22].. Non-dormant seeds can be recognized by the open hilar fissure [22]. The hilum region of seven of the seeds was painted with petroleum gel to obturate the hilar fissure, while in seven seeds the hilum fissure was left open. The remaining six dormant seeds were not treated in any way to serve as a control. Both dormant and non-dormant seeds were placed in an aqueous solution of 25% Aniline Blue, in glass trays with one seed per basin. Seeds were removed at 15 min intervals from the solution. After 15 min to 1h and 30 min, seeds were sectioned longitudinally, by hand, through the hilar pad, along the hilar fissure, to observe the penetration of dye. Observation and imaging of the cross-sections and water gap samples was conducted using a Nikon SMZ1500 stereo-microscope and imaged with a PaxCam Arc digital camera equipped with Pax-it! 2 Version 1.5 software (MIS Inc, Villa Park, IL).

**Character evolution and relationships among seed traits**

Nine categorical and 13 continuous characters (Table 1) were generated based on available *Cuscuta* seed morphological and anatomical literature (Table S1). Description of shapes was based on [42]. Three additional characters consisting of ratios between anatomical continuous features and seed length (Table 1) were added after the initial character scoring. Basic statistics (e.g., averages, standard deviations, normal distribution tests) and Pearson’s correlations were conducted using PAST version 3.16 [43].

**Table 1.** Seed characters surveyed and their representative codes and states. Continuous characters values are averages.

| **Character** | **Character states** |
| --- | --- |
| **Categorical characters** | |
| 1. Compression of seed or the number of ± flat faces that a seed has. | 1 = dorsoventrally compressed = seeds with one flat face and one convex face; 2 = “angled” seeds with 2 flat faces and one convex face; 3 = no compressions, spherical or ovoid |
| 2. Seed shape (considering part with the hilum to be base of the seed) | 1 = elliptic; 2 = obovate; 3 = circular; 4 = ovate, 5 = oblong |
| 3. Radicular end of embryo | 1 = spherically enlarged; 2 = filiform |
| 4. Hilum position | 1 = terminal; 2 = subterminal |
| 5. Hilum compression | 1= flat; 2 = concave |
| 6. Dry seed epidermal cells | 1 = pitted; 2 = non-pitted |
| 7. Hydrated seed epidermal cells | 1 = papillose; 2 = non-papillose |
| 8. Seed epidermis cell shape (as seen in surface SEM images) | 1 = elongated; 2 = isodiametric |
| 9. Presence of outer palisade layer | 0 = absent; 1 = present |
| **Continuous characters** | |
| 10. Number of embryo coils (a “coil” represents a 360° rotation of the embryo) | - |
| 11. Number of seeds per capsule | - |
| 12. Seed length | µm |
| 13. Seed width | µm |
| 14. Seed thickness | µm |
| 15. Hilum area length | µm |
| 16. Hilum area width | µm |
| 17. Length of funicular scar of the hilum | µm |
| 18. Epidermal cell diameter | µm |
| 19. Thickness of epidermal cell (anatomy) | µm |
| 20. Width of epidermal cell (anatomy) | µm |
| 21. Thickness of outer palisade layer (anatomy) | µm |
| 22. Thickness of inner palisade layer(anatomy) | µm |
| **Ratios**  23. Ratio of epidermal cell diameter and seed length. (Subg. Monogynella taxa were not included because of their different epidermal morphology). | - |
| 24. Ratio of epidermal cell thickness and seed length | - |
| 25. Ratio of inner + outer palisade thickness and seed length | - |

Character states were mapped onto a recent genus phylogeny based on *rbcL* and nrLSU [44]. Distribution of characters was analyzed only in-group as the position of *Cuscuta* within Convolvulaceae is currently not resolved [45]. Scenarios of character evolution were analyzed using the parsimony reconstruction method provided by Mesquite 3.40 [46]. Markov k-1 state 1 parameter model (MK1) of evolution was used. In the parsimony reconstruction, character-state changes were treated as unordered. Three qualitative, non-polymorphic characters (outer palisade layer presence, epidermal cell type and type of embryo) were also analyzed with the likelihood reconstruction method [46]. The correlation between the seed epidermal shape and their ability to reverse between pitted and papillate (binary characters, Table 1) was determined using Pagel’s method [47] implemented in Mesquite.

**Number of seed per capsule and breeding systems**

We used the pollen/ovule (P/O) ratio data published by Wright et al. [27]. The latter authors had also assigned taxa to breeding system categories based on Cruden’s ranges [48]: six species were inferred to be fully xenogamous, 108 taxa facultatively xenogamous and at least 23 taxa facultatively autogamous [27]. Differences among P/Os and number of seeds per capsule (S/C) averages were analyzed using an Analysis of Variance (ANOVA). Additionally, a regression tree was constructed (“r.part” – [49]). The defined response variable was the P/O ratio, the explanatory variables the breeding system categories, and the average number of seeds per capsule the prediction model.

**Seed size and distribution range of *Cuscuta* species in North America**

Geographical distribution range size (km^2^) data for 50 North American *Grammica* species were taken from Ho and Costea [15]. As seed length was strongly positively correlated with both the width and thickness of seeds (see Results), it was selected to represent the “seed size” variable. Geographical range size data did not follow a normal distribution [15], and a Spearman’s Rank Correlation with seed size was conducted using PAST version 3.16 [43].

**Seed size, germination and seedling survival**

A comparative seedling survival experiment was conducted in three species, *C. epithymum, C. costaricensis* and *C. campestris*, which have seeds of different sizes [50–52). To corroborate the seed size differences, 500 seeds of each species were imaged and measured using a Nikon SMZ1500 stereomicroscope using Pax-it ver. 1.4.2.0 software and a PaxCam Arc digital camera (MIS Inc., Villa Park, IL). Seed weight was also determined using a Cole-Parmer Symmetry PA - 124I analytical balance. Basic statistics, normality, and a one-way Analysis of Variance (ANOVA) were performed to verify that seed size was significantly different among the three species using PAST version 3.16 [43].

The physical dormancy of *C. campestris* and *C. costaricensis* seeds was removed by a scarification treatment in 99.99% sulfuric acid for 30 minutes, after which seeds were rinsed with sterile Milli-Q water, submerged in bleach for three minutes and then thoroughly rinsed again with sterile water. As the seeds of *C. epithymum* are known to possess a combinational physical and physiological dormancy [8], after the sulfuric acid stratification, they received an additional treatment of gibberellic acid (GA) 1000 ppm [53].

Treated seeds of each species were transferred into sterile 140 mm sterile Petri dishes, each with two Whatman filter paper, moistened with 15 ml of sterile Milli-Q water and incubated at 32˚C and light (150 mmol m^-2^ s^-1^, 12h/day) for germination. Once the tip of the radicle-like organ emerged approximately 1 mm from the seed coat, seeds were considered germinated and were transferred to smaller sterile Petri dishes (90 mm) prepared with one Whatman filter paper and 5 ml sterile Milli-Q water. In total, 150 seedlings per species, distributed two per Petri dish were examined. Petri dishes were sealed with Parafilm M and placed in the greenhouse at 18 °C/21°C, 8/16 h (light intensity 39.6 μmol m^-2^ s^-1^), fully randomized. Seedlings were monitored daily and re-randomized every three days. Sterile Milli-Q water was added throughout the study to maintain the filter paper humid. Seedlings were considered “dead” once the entire seedling was necrotic, from the radicular end to shoot tip. Seedling survival data was analyzed using Kaplan-Meier survival curves with XLSTAT version 2019.4.2.

**Results**

**External morphology and micromorphology; character evolution and correlations**

Characters surveyed are outlined in Table 1 and their complete scoring is presented in Table S2.

Seeds of *Cuscuta* develop within a two-locular ovary, with constantly two anatropous, unitegmic ovules per locule. However, 1–4 mature seeds will develop per capsule (Table S2). As seeds develop in close proximity to one another, the number of seeds per locule determines their compression morphology (Table 1). When seeds adjoin within the same locule, they will possess an “angled” morphology; one seed per locule leads to a dorsoventrally compressed morphology, and one seed per capsule will result in a non-compressed morphology, spherical to ovoid. As the number of seeds varied somewhat within each taxon, from capsule to capsule, two or three compression character states were observed in about 65% of taxa (Table S2). However, the average number of seeds developed per capsule (S/C) was relatively constant in each taxon and varied from 1 to 3.8 (Table S2). Capsules with constantly one seed per fruit evolved in seven *Grammica* clades (Table S2). In general, species of subg. *Cuscuta* averaged the highest S/C (3.1–3.8), and therefore a majority of their seeds were “angled”. Hilum position is also associated with the compression morphology: a lateral hilum is present in species with dorsi-ventrally compressed seeds, while a terminal hilum was observed in “angled” and non-compressed seeds (Table S2). The most common seed shapes encountered were elliptic and ovate, while oblong and circular seeds were observed less frequently; however, most species exhibited a combination of two or three seed shape characters (Table S2).

Epidermis cell shape is always correlated with the ability of seeds to revert between pitted and papillose morphology (Pagel’s test, 5000 simulations, p = 0) and two types of seed epidermises were distinguished:

1. Type I (Fig. 1A) has rectangular, elongated epidermal cells; parallel groups of 2–6 such cells are perpendicular on the long axis of similar groups of cells. Epidermal cells are invariant morphologically, unaffected by dryness and wetness (see the next type). Type I characterizes species of subg. *Monogynella*.
2. Type II (Fig. 1B–F) has more or less isodiametric epidermis cells that can shift their morphology alternating between two states: either pitted (concave) when seeds are dry (Fig. 1B–D), or dome-shaped, papillose (convex) when seeds are hydrated (Fig. 1E–F). This is the most common seed epidermis in dodders, present in the subgenera *Cuscuta, Pachystigma* and *Grammica*.

**Figure 1.** Seed features revealed with scanning electron microscopy. A. Epidermis surface, *C. lupuliformis* (subg. *Monogynella*). B–D. Overall seed morphology and epidermis surface of dry seeds. B. *Cuscuta europaea* (subg. *Cuscuta*). C–D. *Cuscuta cephalanthi* (subg. *Grammica*). C. Entire seed. D. Detail of pitted epidermis of dry seeds. E–F. Different stages of epidermis rehydration. E. *Cuscuta gronovii* var. *gronovii*. F. *Cuscuta cephalanthi*. G–H. Hilum area. G. *Cuscuta approximata*. H. *Cuscuta mitriformis*. I–K. Embryo morphology. I. Filiform and coiled, *C. pacifica*. J–K. Globose toward the radicular end. J. *Cuscuta nevadensis*. K. *Cuscuta microstyla*. L–M. Longitudinal sections through the hilum area showing all the seed components. L. *Cuscuta epithymum*. M. *Cuscuta globulosa*. N–P. Seed coat anatomy. N. *Cuscuta lupuliformis*. O. *Cuscuta alata*. P. *Cuscuta gronovii* var. *gronovii*. Ep = epidermis; En = endosperm; E = Embryo; P1 = Inner or single palisade layer; P2 = Outer palisade layer. Scale bars. A, E, F = 200 μm; D = 40 μm; G, H = 100 μm; B, C, J–M = 0.5 mm; N–P = 50 μm.

Ancestral parsimony reconstruction of the two types of epidermises was equivocal while maximum likelihood reconstruction marginally supported Type I as the ancestral character state (Proportional likelihood Type I: 0.5585; Type II: 0.4414; Fig. 2A). Considering the putative evolutionary advantages provided by Type II for seed imbibition (see Discussions), this is indeed most likely the derived character state.

**Figure 2.** Summary of character evolution hypotheses. A. Invariant epidermis with rectangular, elongated epidermal cells (Type I) is likely ancestral and characterizes subg. *Monogynella*. An epidermis with isodiametric cells that can alternate their morphology between dome-shaped and pitted (Type I) evolved in subgenera *Cuscuta*, *Pachystigma* and *Grammica*. B. Embryo and architecture of palisade layers. The seed coat with only one palisade layer (P1) in subg. *Monogynella* is likely ancestral while a seed coat with two palisade layers (P1 and P2) in the remaining subgenera is likely derived; one palisade layer reverted two times in subg. *Grammica* in *C. microstyla* (clade O) and sect. *Denticulate* (clade E). The latter taxa also evolved an embryo with an enlarged radicular end, which likely functions as a storage organ.

The micropyle is completely obstructed during seed development and no remnants of it were observed in the mature seeds. The hilar area is always a morphologically distinct region, regardless of the type of seed coat. Hilum area is round to elliptic and has in the center the funicular scar or hilar fissure (Fig. 1G–H). Epidermal cells of the hilar pad are substantially smaller than in the rest of the seed epidermis; rectangular-elongated and concentrically arranged around the hilar fissure (Fig. 1G–H). Length, width, and size values of hilar fissure were the highest in subg. *Monogynella* — in some species three to four times larger than in remaining subgenera (Table S2). Although decreasing in size in subg. *Grammica*, Clades D (sect. *Oxycarpae*) and G (sect. *Lobostigmae*), had also relatively large hilar pads and fissures (Table S2).

In general, there are several degrees of variation among taxa across the genus in regard to the quantitative characters (e.g., seed length, hilar pad size), however within each taxon, the variation was relatively consistent (Table S3). For example, seed length ranged from 704.55 µm to 3158.30 µm, while width varied from 666.28 µm to 2910.5 µm (Table S3). Seed size within species had a standard deviation of as little as 16.87 µm (*C. membranacea*) to as much as 196.2 µm (*C. monogyna*), indicating that seed size is a relatively reliable character within each species. Seeds of subg. *Monogynella* are the largest, whereas those of subg. *Cuscuta* are the smallest (Table S2). Subgenus *Grammica* species exhibit the most extensive variation of seed size, for example taxa in Clade G (sect. *Lobostigmae*) had an average seed length of 1658 µm and width of 1420 µm, while taxa of Clade L (sect. *Umbellatae*) had a seed length of 972 µm and width of 846 µm (Table S2). Seed length was strongly correlated with seed width and thickness (Table S4). Similarly, the length of the hilum area was strongly correlated with its width and the length of the funicular scar (Table S4).

When mapped into the genus phylogeny, all the quantitative characters were polymorphic and exhibited extensive homoplasy. For example, subg. *Grammica* taxa of Clade D (sect. *Oxycarpae*) and Clade G (sect. *Lobostigmae*) have evolved similar seed length, epidermal cell thickness, inner and outer palisade thickness, whereas, taxa of Clade A (sect. *Californicae*) and Clade L (sect. *Umbellatae*) have similar hilar pad length and width and palisade layer(s) thickness.

**Anatomy of seed coat; character evolution and relationships among characters**

The seed coat originates from the single ovule integument and has a simple structure, consisting of a two or three cell layered testa and several crushed parenchymatic cells representing the tegmen. The embryo is filiform, coiled within the endosperm.

Seen in longitudinal sections, Type I epidermal cells (of subg. *Monogynella*) appear more or less rectangular and contain abundant tannins. Type II epidermal cells (of subgenera *Grammica, Cuscuta* and *Pachystigma*) are radially elongated, tapered basally and rounded distally. Developing seeds had starch grains in their epidermis cells. The ratio between the epidermal cell thickness and the seed size was largest in subg. *Cuscuta* and the smallest in subg. *Monogynella* (Table S2).

The majority of *Cuscuta* species possess an inner and an outer palisade layer, which are continuous (“complete”) throughout the entire seed coat, including in the hilar area (Fig. 1L–M; O–P). As an exception, in subg. *Monogynella* and four species of subg. *Grammica* (see below), the outer palisade layer is “incomplete”, present only under and around the hilar region, and absent from the rest of the seed coat (where only a single palisade layer can be observed; Fig. 1N; Fig. 2B). The double palisade layer originates from a periclinal division of the same cell layer that serves as an origin for the single palisade layer architecture. Palisade layer(s) of immature seeds possess thin cellulosic cell walls and contain abundant starch grains (Fig. 3I). The inner and single palisade layers cells undergo a secondary thickening through deposition of lignin, which obturates their lumen almost entirely (Fig. 1N–P; Fig. 3 H, J, K). Thus, the inner palisade layer (in case of the double palisade architecture) and the single palisade layer (for the single architecture) are homologous. These palisade layer cells exhibit a linea lucida (or “light line”), a light refractive, apparently denser region in the upper third of radial cell walls (Fig. 3H, J, K). The outer palisade layer (in the case of the double palisade architecture) cells are shorter than those of the inner palisade layer; they do not exhibit a light line and their cells walls remain relatively thin, although also lignified (Fig. 1O–P). As palisade layer(s) represent(s) the mechanical layers, based on the classification of [54], it results that *Cuscuta* seeds are endotestal.

**Figure 3.** Seed features viewed with light microscopy. A–D. Embryos. A. *Cuscuta monogyna* (embryo removed from the endosperm). B. Embryo of *C. tinctoria* var. *floribunda* embedded in the endosperm. C. Developing embryo of *C. nevadensis* surrounded by the endosperm epidermis (the rest of endosperm was nearly entirely consumed). D. Fully developed embryo of *C. nevadensis* (endosperm epidermis removed). E–H. *Cuscuta lupuliformis* (subg. *Monogynella*). E–G. Longitudinal sections through the hilum area of *C. lupuliformis*. Arrows indicate water gap with tracheid-like structures. H. Testa architecture with only one palisade layer. I–K. Seed coat architecture with two palisade layers. I. Incipient stage in the development of two palisade layers in *C. argentinana*; at this stage epidermis contains starch grains. J. *Cuscuta europaea*. K. *Cuscuta cristata*; note the presence of linea lucida in inner palisade layer (P1). I. Parenchyma cells with lipids and starch in the enlarged portion of *C. nevadensis* embryo. M. Longitudinal section of rehydrated *C. sandwichiana* seed after 30 min of in Aniline blue; dye penetration is limited to the water gap (indicated with arrows). E = Embryo; En = endosperm; H = hilum; Ep = epidermis; P1 = Inner or single palisade layer; P2 = Outer palisade layer. Scale bars. A–F = 0.5 mm; G, I–K = 50 μm; H, L = 25 μm; M = 100 μm.

Likelihood reconstruction marginally supported a derived status for the double palisade layer from the single palisade architecture (proportional likelihood = 0.5586; Fig. 2B), which is also suggested by the ontogeny of the former. Reversals to a single palisade layer anatomy have occurred two times in four species of two clades within subg. *Grammica*: all the species of sect. *Denticulatae* (Clade E; *C. denticulata, C. nevadensis* and *C. veatchii*) and *C. microstyla* in sect. *Subulatae* (Clade O) (Fig. 2B).

The hilar pad epidermis cells are small, rectangular and thin-walled, cellulosic. As indicated above, the seed coat structure within the hilar pad is invariant across the entire genus being always composed of two palisade layers. Especially the inner palisade layer increases significantly in thickness (up two times) in the hilum area compared to its size in the rest of the seed coat. A suture-type discontinuity within the epidermis and palisade layers at the centre of the hilar pad forms the hilar fissure (Fig. 3E, F). This is also where the seed water gap is located. Tracheids, which are most likely remnants of the funiculus vasculature, were observed in this region (Fig. 3G). The dye tracking experiment revealed that although the epidermis cells of dormant seeds hydrated (which can be determined by their bulging and absorbing of stain), the aniline blue solution did not penetrate through the palisade layer(s) even 60 min after soaking in the dye. In contrast, in non-dormant seeds, the dye began to infiltrate through the hilar fissure after 15 min. After 120 min, the stain was observed around the endosperm and embryo of non-dormant seeds (Fig. 3M). The dye also infiltrated into the endosperm and embryo via irregular fissures within the palisade layers caused by accidental mechanical injury during processing.

The incipiently developing endosperm is nuclear and many free nuclei were observed; however, eventually cell wall formation is initiated and gradually progresses centripetally. Endosperm of young seeds is starchy and becomes “gelatinous” in mature seeds. Gelatinization is apparent only in hydrated seeds; dry seeds have a “hard” endosperm. A peripheral, “membranous”, cell layer with large nuclei (called aleurone layer by [12, 20]) was observed around the endosperm, demarcating it from the parenchymal layers of the tegmen. Ten species of subgenera *Grammica* and *Pachystigma (Grammica: C. sandwichiana* - Clade B (sect. *Racemosae)*; *C. nevadensis, C. denticulata, C. veatchii* - Clade E (sect. *Denticulatae*); *C. haughtii* – Clade F (sect. *Partitae*); *C. tinctoria* – Clade G (sect. *Lobostigmae*); *C. strobilacea* – Clade K (sect. *Ceratophorae*); *C. acuta* – Clade L (sect. *Umbellatae*); *C. microstyla* – Clade O (sect. *Subulatae*); *Pachystigma: C. nitida)* displayed a markedly thicker endosperm epidermis, which separated easily from the rest of the seed coat.

The embryo is most commonly filiform, devoid of meristems at the radicular end, and with no cotyledons. The number of embryo coils varies; in some species it appears to curve resembling a cane, but no coils form (Fig. 3A), while in others the embryo displays anywhere from one to four and half coils (Figs. 1A, 3 B, C; Table S2). The number of coils varies considerably amongst subgenera, but remains relatively consistent within individual species. Subgenera *Monogynella*, *Pachystigma* and *Cuscuta* possess the lowest number of coils (1–2), while an increased number of coils has evolved multiple times in subg. *Grammica* (Fig. S1). A peculiar embryo, spherically enlarged toward the radicular end has evolved in four species of subg. *Grammica: C. denticulata*, *C. veatchii*, *C. nevadensis* (Clade E – sect. *Denticulatae* Fig. 1J; 3C, D) and in *C. microstyla* (Clade O – sect. *Subulatae*; Fig. 1K). These taxa exhibit a spherical swelling toward the radicular portion of the embryo, differing only in the size of the globose part and the number of coils in the filiform part toward the shoot (between zero and 1.5 coils).

The endosperm of these taxa is much reduced compared to other *Cuscuta* species, limited to several marginal cell layers which are entirely consumed during embryo development in such a way that when seeds are mature, the endosperm is represented only by its epidermis. The globose radicular part consists of an epidermis and a storage parenchyma with starch and lipid droplets (Fig. 3L). It should be noted that this remarkable embryo morphology is associated in these species with the reduction of the testa to a single palisade layer (Fig. 2B). This type of embryo has clearly evolved from the ancestral filiform embryo characteristic for the remainder of the genus (Fig. 2B).

**Breeding systems and number of seeds per capsule in *Cuscuta***

ANOVA indicated a significant relationship between the number of seeds per capsule (S/C) and the breeding system categories. Fully autogamous species had, on average, the highest number of seeds per capsule, whereas fully xenogamous taxa had the lowest (Fig. 4). In the regression tree, the first split separated a leaf of 14% facultative autogamous taxa from the remainder of species, followed by additional splits based on their S/C averages (Fig. 5). The terminal leaves of these additional splits divided the remaining 86% of taxa into additional leaves illustrating a steady increase of P/O values as the S/C average decreased (Fig. 5).

**Figure 4.** Analysis of variance for the breeding system categories and the average number of seeds per capsule (S/C). A. Facultatively autogamous taxa have the highest S/Cs averages, but also the highest variation. B. Facultatively xenogamous group include species that possess an intermediate S/C number between the other two categories. C. Fully xenogamous taxa have the lowest S/C average and the least amount of variation; species in this category are self-incompatible.

**Figure 5**. Regression tree analysis of number of seeds per capsule (NRSeedCapsule) and pollen/ovule ratios (P/O) used as an indicator of breeding systems. The first split separated directly the leaf of 14% facultatively autogamous taxa with an average P/O of 226 (first leaf to the left). At the next node, the remainder species were divided depending on whether they had more or less than 2.5 S/C. 45% of taxa had more than 2.5 S/C and were split again depending whether they had more or less than 3.3 S/C. 14% of taxa had more than 3.3 S/C and were placed in the second terminal leaf, with a P/O of 746.31% had less than 3.3 S/C and were separated in the third terminal leaf, with a P/O of 1010. Taxa with less than 2.3 S/C were found in the sixth terminal leaf, comprising 11% of the total, P/O of 1681. Taxa with more than 2.3 S/C were divided once more if they have more of less than 1.3 S/C. **7%** of the total had less than 1.3 S/C, P/O 1012, while 22% had more than 1.3 S/C and P/O of 1369. NRSeedCapsule **=** Number of seeds per capsule.

**Seed size and distribution range of *Cuscuta* species in North America**

Spearman’s Rank correlation indicated a lack of correlation between the seed size and the total geographical distribution range of species (r = 0.0944, $r^{2}$= 0.0089). This strongly suggests that seed size does not have an impact on the dispersal and the total geographical range of the species in N America.

**Seedling survival**

There was a significant difference in seed size among the three species studied (Table S5). Kaplan-Meier seedling survival curves showed that seedlings originating from larger-sized seeds survived significantly longer than seedlings of smaller-sized seeds (Fig. 6). The seedlings of *C. campestris*, having the largest seeds among the three species examined, survived longer (48.12 days) than the seedlings of *C. costaricensis* (36.79 days) and *C. epithymum* respectively (20.49 days).

**Figure 6.** Kaplan-Meier survival curve showing the proportion of *Cuscuta* seedlings surviving (log scale). Species are listed in order of their increasing seed size: blue line = *C. epithymum*, the smallest seeds (0.89 mm); red line = *C. costaricensis*, intermediate sized-seeds (1.08 mm); green line = *C. campestris*, the largest seeds (1.28 mm). Standard error not shown for clarity.

**Discussion**

*Potential use of seed characters for systematics and identification of Cuscuta species*

This is the most comprehensive study of *Cuscuta* seeds to date, examining for the first time 80 species and filling either morphological or anatomical knowledge gaps for the 21 previously studied species (Table S1). Our findings largely confirmed previous studies (Table S1), as well as earlier taxonomic revisions in which seeds were superficially described (mostly in terms of size and sometimes shape [e.g., 16; 17, 51, 52, 55–65]). Despite the significant increase in taxon sampling size and the fine level of morphological and anatomical detail employed, only a few additional features were discovered. Species of subg. *Monogynella* are distinct compared to the rest of the genus because of their singular epidermal surface morphology, larger size, and one-palisade layer architecture of testa. The remaining subgenera of *Cuscuta* can also be distinguished for most species using a combination of morphological and anatomical traits (Table S2). The 15 sections of subg. *Grammica* [1], however, cannot be separated because of the high level of homoplasy observed (although some exceptions exist; e.g., sect. *Denticulatae*). Although not systematically significant at an infrageneric sectional level, seed morphology and anatomy can provide valuable taxonomic data for identification purposes. As indicated by other authors (e.g., [9, 12, 20], species identification is difficult by seed characters alone, but not impossible if the geographical origin of seeds is known which reduces the number of potential species from among which the identification starts. If the geographical origin of seeds is unknown, identification can be narrowed down to subgenus, and species recognition can be completed using a molecular approach using the sequences we have uploaded in Genbank for numerous species. In this latter case, we advise caution as many of the sequences uploaded in Genbank may have originated from misidentified plants (see the discussions in [52, 64]) and the systematics of many *Cuscuta* clades is still unresolved at a species level.

**Evolution of form and function in *Cuscuta* seeds**

Several studies [e.g., 21, 66] indicated that similarities exist between the surface of the seed coat in *Cuscuta* and other genera in Convolvulaceae (e.g., *Convolvulus*, *Cressa*, *Evolvulus*, *Ipomoea*, and *Seddera*). Despite the fact that the epidermal cells of some Convolvulaceae taxa are isodiametric and more or less dome-shaped, resembling those of *Cuscuta* (e.g., [67--69]), it is unknown if they can alternate from pitted to papillose depending on their hydration status. This interesting trait observed in all the *Cuscuta* species with the exception of subg. *Monogynella*, is apparently ensured by flexible nature of the outermost cell walls of the epidermal cells [12, 19, 70] and the presence of hydrophilic pectic zones, capable of attracting and retaining water [12, 20, 70].

[20] and [71] suggested that the pitted epidermis morphology of *Cuscuta* dry seeds is an adaptation for wind dispersal. However, dodder seeds are much larger than typical anemochory adapted seeds like those of Orobanchaceae or some Plantaginaceae, which are “dust-like” and exhibit clearly reticulate or “honey-comb” epidermal morphologies [72–74]. Based on the results of this study, it is more likely that the pitted/papillate seed coat is an adaptation related to the imbibition, and more broadly, germination. The presence of pectin in the cuticle and epidermal cell walls results in the creation of a mucilaginous layer around the seeds when seeds are hydrated [6, 20, 75], protecting them from desiccation [76] and allowing them to adhere to soil particles. This epidermis trait also allows seeds to be dispersed via farm machinery [6] and bird epizoochory (e.g., [77]). Last but not least, the hydrated epidermis prevents the displacement of seeds within the soil profile, which is important because *Cuscuta* species require light for germination [78–81]. These functional advantages of the pitted/papillose seed epidermis support the result of the likelihood ancestral reconstruction according to which this trait is derived from the “regular” epidermis encountered in subg. *Monogynella*.

[54] supported the classification of *Cuscuta* in Convolvulaceae based on the ontogeny and structure of the seed coat, which is shared by all the studied family members [68, 82, 83]. The inner palisade (or single palisade layer of subg. *Monogynella* and several *Grammica* species) contains a light line (linea lucida) similar to that present in the seeds of other taxa with impermeable seed coats (e.g., Convolvulaceae, Cucurbitaceae, Fabaceae, Geraniaceae, Malvaceae; reviewed by [84]). An impermeable palisade cell layer(s) or “hardseedness” has been associated with physical dormancy in many angiosperms, including in *Cuscuta* and *Convolvulaceae* [84–88]. In *Cuscuta*, the inner (or single palisade when only palisade layer is present) is strongly lignified and it represents the ultimate mechanical defense of the seeds. Bird endozoochory experiments reported that gut passage partially or entirely stripped the outer palisade layer, while the inner or single palisade layer was little affected [13, 14]. If the function of the inner palisade layer (or single one in *Monogynella*) is to safeguard the impermeability of the seed coat, ensure physical dormancy and provide an ultimate line of mechanical defense, the role of the external palisade layer is not clear. The cells of the external palisade layer are in close contact with the epidermal cells, and in addition to protection, they may be involved in the opening of the hilar water gap.

Current findings showed that the outer palisade layer can be lost through evolution in the *Grammica* species with a globose embryo. All the above mentioned subtle adaptations for dispersal, e.g., via epi- or endozoochory, indicate that the characterization of *Cuscuta* seeds as “unspecialized” [e.g., 10–12] is not suitable (see also the discussion in [14]).

Originally, it was thought that the papillae on *Cuscuta* seed epidermis represented the access point of water to the embryo [20, 79]. Our results confirmed the findings of [22] in *C. australis,* in that the water enters into intact seeds through the hilar fissure/water gap. We found that the water gap anatomy in *Cuscuta* is uniform despite variations observed in the structure of the testa (e.g., epidermis type and number of palisade layers). In other Convolvulaceae seeds (e.g., *Ipomoea, Merremia, Calystegia*) “bulges” adjacent to the micropyle initiate the water entry into the seed [41, 89]. In *Cuscuta*, the opening of the hilar fissure during imbibition may be the result of the overall tensions created within the palisade layer(s) (particularly the external one) by the turgescent epidermal cells.

Many authors have mentioned or investigated the development of the filiform, coiled embryo in *Cuscuta* [12, 20, 79, 90–94]. The coiling of the embryo has been said to foreshadow the parasitic behavior of the plant as it coils around its host [10]. It is more conceivable, as [94] suggested, that the coiling pattern allows for a longer embryo to develop within the limited space of the seed, and upon germination, the extra length gained through the straightening of the coils may represent an advantage for the seedlings that need to elongate rapidly in order to locate a host. *Grammica* species of sect. *Denticulatae* and *C. microstyla* (sect. *Subulatae*) evolved an embryo with an enlarged spherical or club-shaped radicular end. The species of sect. *Denticulatae* [56, 95] are viviparous: their seeds germinate while still found inside the capsules and the parasite is attached to the host. This ensures that the seedlings will be able to attach directly to the shoots of same host plant. Considering the desert habit these species occur in [56, 95], it is most likely that this peculiar embryo has evolved as a storage organ. Less is known about the natural history of *C. microstyla* (section *Subulatae*), but this species also grows in arid habitats in the Andes [56, 96].

**Allometric relationships of seeds and seedling survival**

We have confirmed that autogamous species had the highest number of seeds per capsule (S/C) whereas fully xenogamous taxa had the lowest. Having a mixed mating system, allows *Cuscuta* species to combine in different proportions the reproductive assurance of selfing with the boost of genetic diversity provided outcrossing [27, 97, 98]. High measures of reproductive output (e.g., seed/ovule ratio, number of seed/fruit) characterize annuals, while these measures are generally lower for perennials, which are more often outcrossing or clonal [e.g., 99–102]. *Cuscuta* species are usually considered to be annual [e.g., 3, 4, 8, 19]. However, many species growing on perennial herb hosts and especially woody hosts behave as perennial because they can regenerate yearly from haustorial tissue left inside the host [6, 8, 103–106]. We have often noted in the field in Mexico that *Cuscuta* species characterized as fully xenogamous by [27] (e.g., *C. volcanica*) are parasitic on woody plants and “perennial” from an endophyte, while facultatively autogamous dodders grow on annual hosts and synchronize their life cycle with them. This potential relationship has not been studied to date, but considering that most weedy and invasive dodders comport as annual [3, 4, 6], a possible connection between the seed production, breeding system and host range would be interesting to investigate in the future.

Seed size is strongly related some plants with their dispersal ability [107--109], but in *Cuscuta* we have found no indication of such a relationship. Using the same geographical dataset, [15] reported that distribution patters of subg. *Grammica* in N America are strongly associated with the dehiscence or indehiscence of capsules. As the current results suggested, it is more likely that seed size investment in *Cuscuta* is related to the seedling survival rather than with the dispersal capability.

In many other plants, seed size has been shown to be positively correlated with higher seedling survivorship rates when seedlings face unfavorable conditions (e.g., drought, deep shade, high depth within the soil) because larger seeds have more food reserves [e.g., 110–112]. In the case of *Cuscuta*, the seedling stage ontogenetic bottleneck is even more critical because in addition to surviving abiotic and biotic challenges similar to green plants [e.g., 35, 113–115] , seedlings must also locate and overcome within a limited amount of time the defenses of compatible hosts [e.g., 4, 6]. Seed size affects seedling survival time because seedlings are unable to photosynthesize and thus they depend entirely on their seed reserves. The long survival times reported in this study, 40 to 90 days depending on the species, are unlikely to be found under natural conditions because seedlings were fully protected against desiccation and “death” was noted only when seedlings were entirely necrotic. Survival times ranging from one to several weeks are more likely to be found under natural conditions as reviewed by [36].

**Acknowledgments**

Catherine Swytink-Binnema and Hiba El Miari helped with the preparation of some of the SEM samples. Ádám Lovas-Kiss kindly provided one of the samples of *C. lupuliformis*. Last but not least, we thank two anonymous reviewers for their suggestions.

**References**

1. Costea M, García MA, Stefanović S. A phylogenetically based infrageneric classification of the parasitic plant genus *Cuscuta* (dodders, Convolvulaceae). Syst Bot. 2015; 40: 269–285.
2. Press MC, Phoenix GK. Impacts of parasitic plants on natural communities. New Phytol. 2005; 166: 737--751.
3. Parker C, Riches CR. Parasitic weeds of the world. Biology and control. Wallingford: CAB International; 1993.
4. Dawson JH, Musselman LJ, Wolswinkel P., Dörr I. Biology and control of Cuscuta. Rev Weed Sci. 1994; 6: 265-317.
5. Holm L, Doll J, Holm E, Pancho JV, Herberger JP. World weeds: natural histories and distribution. John Wiley & Sons; 1997.
6. Costea M, Tardif FJ. The biology of Canadian weeds. 133. *Cuscuta campestris* Yuncker, *C. gronovii* Willd. ex Schult., *C. umbrosa* Beyr. ex Hook., *C. epithymum* (L.) L. and *C. epilinum* Weihe. Can J Pl Sci. 2006 86: 293--316.
7. Sandler HA. Managing *Cuscuta gronovii* (swamp dodder) in cranberry requires an integrated approach. Sustainability. 2010; 2: 660--683.
8. Meulebrouck K, Ameloot E, Van Assche JA, Verheyen K, Hermy M, Baskin CC. Germination ecology of the holoparasite *Cuscuta epithymum*. Seed Sci Res. 2008; 18: 25--34.
9. Knepper DA, Creager RA, Mussleman LJ. Identifying dodder seed as contaminants in seed shipments. Seed Sci Technol. 1990; 18: 731--741.
10. Kuijt J. The biology of parasitic flowering plants. Berkley, California: University of California Press, 1969.
11. Musselman LJ. Parasitic weeds and this impact in Southwest Asia. Proc Royal Soc Edinburgh. 1986; 89B: 283--288.
12. Lyshede OB. Studies on mature seeds of *Cuscuta pedicellata* and *C. campestris* by electron microscopy. Ann Bot. 1992; 69: 365--371.
13. Costea M, Stefanović S, García MA, De La Cruz S, Casazza ML, Green AJ. Waterfowl endozoochory: An overlooked long‐distance dispersal mode for *Cuscuta* (dodder). Am J Bot. 2016; 103: 957–962.
14. Costea M, El Miari H, Laczkó L, Fekete R, Molnár AV, Lovas-Kiss Á, Green AJ. The effect of gut passage by waterbirds on the seed coat and pericarp of diaspores lacking “external flesh”: Evidence for widespread adaptation to endozoochory in angiosperms. PLoS One. 2019; 14(12).
15. Ho A, Costea M. Diversity, evolution and taxonomic significance of fruit in Cuscuta (dodder, Convolvulaceae); the evolutionary advantages of indehiscence. Perspect Plant Ecol Evol Syst. 2018; 32: 1--17.
16. Costea M, Stefanović S. *Cuscuta jepsonii* (Convolvulaceae): An invasive weed or an extinct endemic? Am J Bot. 2009a: 96: 1744--1750.
17. Yuncker TG. The genus *Cuscuta*. Mem Torr Bot Club. 1932; 18: 113--331.
18. Engelmann G. Systematic arrangement of the species of the genus *Cuscuta*, with critical remarks on old species and descriptions of new ones. Trans Acad Sci St*.* Louis. 1859; 1: 453--523.
19. Gaertner EE. Studies of seed germination, seed identification, and host relationships in dodders, *Cuscuta* spp. Mem Cornell Univ Agri Exp Station. 1950; 294.
20. Lyshede OB. Seed structure and germination in *Cuscuta pedicellata* with some notes on *C. campestris*. Nordic J Bot. 1984; 4: 669--674.
21. Abdel Khalik KN. Seed morphology of *Cuscuta* L. (Convolvulaceae) in Egypt and its systematic significance. Feddes Repert.2006; 117: 217--224.
22. Jayasuriya KM, Baskin JM, Geneve RL, Baskin CC, Chien CT. Physical dormancy in seeds of the holoparasitic angiosperm *Cuscuta australis* (Convolvulaceae, Cuscuteae): Dormancy-breaking requirements, anatomy of the water gap and sensitivity. Ann Bot. 2008; 102: 39--48.
23. Rodriguez-Pontes M. Seed formation and pollination system in *Cuscuta obtusiflora*: First record of preanthesis cleistogamy in parasitic plants and some functional inferences. Flora. 2009; 204: 228--237.
24. Martinčová M, Kaštier P, Krasylenko YA, Gajdoš P, Čertík M, Matušíková I, Blehová A. Species-specific differences in architecture and chemical composition of dodder seeds. Flora. 2019; 256: 61--68.
25. Stefanović S, Kuzmina M, Costea M. Delimitation of major lineages within *Cuscuta* subgenus *Grammica* (Convolvulaceae) using plastic and nuclear DNA sequences. Am J Bot. 2007; 94: 568--589.
26. Wright M, Welsh M, Costea M. Diversity and evolution of the gynoecium in *Cuscuta* (dodders, Convolvulaceae) in relation to their reproductive biology: Two styles are better than one. Pl Syst Evol. 2011; 296: 51--76.
27. Wright M, Ianni MD, Costea M. Diversity and evolution of pollen-ovule production in *Cuscuta* (dodders, Convolvulaceae) in relation to floral morphology. Pl Syst Evol. 2012; 298: 369--389.
28. Haig D, Westoby M. On limits to seed production. Amer Nat. 1988; 131: 757--759.
29. Larson BM, Barrett SC. A comparative analysis of pollen limitation in flowering plants. Biol J Linn Soc. 2000; 69: 503--520.
30. Ashman TL, Knight TM, Steets JA, Amarasekare P, Burd M, Campbell DR et al. Pollen limitation of plant reproduction: ecological and evolutionary causes and consequences. Ecology. 2004; 85: 2408--2421.
31. Westoby M, Rice B, Howell J. Seed size and plant growth as factors in dispersal spectra. Ecology. 1990; 71: 1307--1315.
32. Westoby M, Leishman M, Lord J. Comparative ecology of seed size and dispersal. Philos Trans R Soc B Biol.1996; 351: 1309-1318.
33. Gaston KJ. The structure and dynamics of geographic ranges*.* London, UK: Oxford University Press; 2003.
34. Kidson R, Westoby M. Seed mass and seedling dimensions in relation to seedling establishment. Oecologia. 2000; 125: 11--17.
35. Moles AT, Westoby M. Seedling survival and seed size: a synthesis of the literature. J Ecol. 2004; 92: 372--383.
36. Behdarvandi B, Guinel FC, Costea M. Differential effects of ephemeral colonization by arbuscular mycorrhizal fungi in two *Cuscuta* species with different ecology. Mycorrhiza 2015; 25: 573--585.
37. Spisar K. Beiträge zur physiologie der *Cuscuta gronovii*. Ber Deut Bot Ges. 1910; 28: 329--334.
38. Verdcourt B. *Cuscuta* L. J Ecol. 1948; 36: 356--365.
39. Peterson RL, Peterson CA, Melville LH. Teaching plant anatomy through creative laboratory exercises. NRC Research Press; 2008.
40. Casley‐Smith JR. Some observations on the fixation and staining of lipids. J Microsc. 1963; 81: 235--238.
41. Jayasuriya KG, Baskin JM, Geneve RL, Baskin CC. Morphology and anatomy of physical dormancy in *Ipomoea lacunosa*: identification of the water gap in seeds of Convolvulaceae (Solanales). Ann Bot. 2007; 100: 13--22.
42. Hickey LJ. Classification of the architecture of dicotyledonous leaves. Am J Bot. 1973; 60: 17--33.
43. Hammer Ø, Harper DAT, Ryan PD. PAST-Palaeontological Statistics Software Package for Education and Data Analysis ver.3.15 University of Oslo. 2001. Available from: [https://folk.uio.no/ohammer/past/](about:blank).
44. García MA, Costea M, Kuzmina M, Stefanović S. Phylogeny, character evolution, and biogeography of *Cuscuta* (dodders; Convolvulaceae) inferred from coding plastid and nuclear sequences. Am J Bot. 2014; 101: 670--690.
45. Stefanović S, Olmstead RG. Testing the phylogenetic position of a parasitic plant (*Cuscuta,* Convolvulaceae, Asteridae): Bayesian inference and the parametric bootstrap on data drawn from three genomes. Syst Biol. 2004; 53: 384--399.
46. Maddison WP, Maddison DR. Mesquite: A modular system for evolutionary analysis. Version 3.61. 2019. Available from: [http://mequiteproject.org](about:blank).
47. Cruden RW. Pollen-ovule ratios: A conservation indicator of breeding systems in flowering plants. Evolution. 1977; 31: 32--46.
48. Pagel M. Detecting correlated evolution on phylogenies: a general method for the comparative analysis of discrete characters. Proc Royal Soc B. 1994; 255: 37--45.
49. McCullagh P, Nelder JA. Generalized linear models. London: Chapman and Hall., 1989.
50. García MA. *Cuscuta*. In: Talavera S et al. editors. Flora Ibérica. Vol. XI: Gentianaceae Boraginaceae. Madrid: Consejo Superior de Investigaciones Científicas; 2011. p. 292--310.
51. Costea M, García Ruiz I, Stefanović S. Systematics of “horned” dodders: phylogenetic relationships, taxonomy, and two new species within the *Cuscuta chapalana* complex (Convolvulaceae). Botany. 2011a; 89: 715--730.
52. Costea M, García MA, Baute K, Stefanović S. Entangled evolutionary history of *Cuscuta pentagona* clade: A story involving hybridization and Darwin in the Galapagos. Taxon. 2015; 64: 1225--1242.
53. Frankland B. Effect of gibberellic acid, kinetin and other substances on seed dormancy. Nature. 1961; 192: 678--679.
54. Corner EJH. The Seeds of Dicotyledons. Cambridge: Cambridge University Press; 1976.
55. Hunziker A T. Las especies de *Cuscuta* (Convolvulaceae) de Argentina y Uruguay (Continuacion). Rev Fac Cienc Exactas Fís Nat. 1950; 13: 177–251.
56. Costea M, Nesom GL, Tardif FJ. Taxonomic status of *Cuscuta nevadensis* and *C. veatchii* (Convolvulaceae) in North America. Brittonia. 2005; 57: 264--272.
57. Costea M, Nesom GL, Stefanović S. Taxonomy of the *Cuscuta pentagona* complex (Convolvulaceae) in North America. Sida. 2006a; 22: 151-175.
58. Costea M, Nesom GL, Stefanović S. Taxonomy of the *Cuscuta indecora* (Convolvulaceae) complex in North America. Sida. 2006b; 22: 209--225.
59. Costea M, Nesom GL, Stefanović S. Taxonomy of the *Cuscuta salina-californica* complex (Convolvulaceae). Sida. 2006c: 22 176-195.
60. Costea M, Aiston F, Stefanović S. Species delimitation, phylogenetic relationships, and two new species in the *Cuscuta gracillima* complex (Convolvulaceae). Botany. 2008; 86: 670--681.
61. Costea M, Stefanović S. Molecular phylogeny of *Cuscuta californica* complex (Convolvulaceae) and a new species from New Mexico and Trans-Pecos. Syst Bot. 2009b; 34: 570--579.
62. Costea M, Wright MA, Stefanović S. Untangling the systematics of salt marsh dodders: Cuscuta pacifica a new segregate species from *Cuscuta salina* (Convolvulaceae). Syst Bot. 2009; 34: 787--795.
63. Costea M, Stefanović S. Evolutionary history and taxonomy of the *Cuscuta umbellata* complex (Convolvulaceae): Evidence of extensive hybridization from discordant nuclear and plastid phylogenies. Taxon. 2010; 59: 1783--1800.
64. Costea M, Spence I, Stefanović S. Systematics of *Cuscuta chinensis* species complex (subgenus *Grammica*, Convolvulaceae): evidence for long-distance dispersal and one new species. Org Divers Evol. 2011b; 11: 373--386.
65. Costea M, García-Ruiz I, Dockstader K, Stefanović S. More problems despite bigger flowers: Systematics of *Cuscuta tinctoria* clade (subgenus *Grammica*, Convolvulaceae) with description of six new species. Syst Bot. 2013; 38: 1160--1187.
66. Hamed KA. Pollen and seed characters of certain *Cuscuta* species growing in Egypt with a reference to a taxonomic treatment of the genus. Int J Agric Biol. 2005; 7: 325--332.
67. Sripleng A, Smith FH. Anatomy of the seed of *Convolvulus arvensis*. Am J Bot. 1960; 47: 386--392.
68. Hamed KA, Mourad MM. Seed exomorphic and anatomical characters of some species of Convolvulaceae. Egyptian J Bot. 1994; 34:1--16.
69. Ketjarun K, Staples GW, Swangpol SC, Traiperm P. Micro-morphological study of *Evolvulus* spp.(Convolvulaceae): the old world medicinal plants. Bot Stud. 2016; 57: 25.
70. Lyshede O. Ultrastructural features of seed and seedling of *Cuscuta* *pedicellata*. Micron Microsc Acta. 1990; 21: 163--164.
71. Koch L. Die Klee-und Flachsseide (*Cuscuta epithymum* und *C. epilinum*): Untersuchungen über deren Entwicklung, Verbreitung und Vertilgung. Carl Winters Universitätsbuchhandlung, 1880.
72. Chang T-I, Heckard LR. Morphology in *Cordylanthus* (Scrophulariaceae) and its taxonomic significance. Am J Bot. 1972; 59: 258--265.
73. Elisens WJ, Tomb SA. Seed morphology in New World *Antirrhineae* (Scrophulariaceae): Systematic and phylogenetic implications. Pl Syst Evol. 1983; 142: 23-47.
74. Eriksson O, Kainulainen K. The evolutionary ecology of dust seeds. Perspect Plant Ecol Evol Syst. 2011; 13: 73--87.
75. Grubert M. Bestimmung des Schleimgehaltes myxospermer Diasporen verschiedener Angiospermenfamilien. Pl Syst Evol. 1982; 141: 7--21.
76. Harper JL, Benton RA. The behaviour of seeds in soil: The germination of seeds on the surface of water supplying substrate. J Ecol. 1966; 54: 151--166.
77. Ridley HN. The dispersal of plants throughout the world. L. Reeve & Company, Ltd; 1930.
78. Allred KR, Tingey DC. Germination and spring emergence of dodder as influenced by temperature. 1964; Weeds 12: 45--48.
79. Hutchison JM, Ashton FM. Effect of desiccation and scarification on the permeability and structure of the seed coat of *Cuscuta campestris*. Am J Bot. 1979; 66: 40--46.
80. Benvenuti S. Soil texture involvement in germination and emergence of buried weed seeds. Agron J. 2003; 95: 191--198.
81. Benvenuti S, Dinelli G, Bonetti A, Catizone P. Germination ecology, emergence and host detection in *Cuscuta campestris.* Weed Res. 2005; 45: 270--278.
82. Govil CM, Lavania S. Floral anatomy and embryology of some species of *Cuscuta L.* Proc. Indian Acad Sci. 1980; 89: 219--228.
83. López-Curto L, Marquez-Guzmán J, Laguna-Hernández G, Ponce-Salazar M. Life cycle and seed development of *Ipomea* x *leucantha* (Convolvulaceae), a weed of rice. Phyton. 1990; 51: 19--24.
84. Baskin JM, Baskin CC, Li X. Taxonomy, anatomy and evolution of physical dormancy in seeds. Plant Spec Biol. 2000; 15: 139--152.
85. Barton LV. Dormancy in seeds imposed by the seed coat. In: Lang A, editor. Differentiation and Development. Berlin, Heidelberg: Springer; 1965. p 2374--2392.
86. Rolston MP. Water impermeable seed dormancy. Bot Rev. 1978; 44: 365--396.
87. Werker E. Seed dormancy as explained by the anatomy of embryo envelopes. Israel J Bot. 1980; 29: 22--44.
88. Kelly KM, Van Staden, J, Bell WE. Seed coat structure and dormancy. Plant Growth Regul. 1992; 11: 201--209.
89. Geneve RL, Baskin CC, Baskin JM, Jayasuriya KMG, Gama-Arachchige NSG. Functional morpho-anatomy of water-gap complexes in physically dormant seed. Seed Sci Res. 2018; 28: 186--191.
90. Hooker HE. On *Cuscuta gronovii*. Bot Gaz. 1889; 14: 31--37.
91. MacPherson GE. Comparison of development in dodder and morning glory. Bot Gaz. 1921; 71: 392--398.
92. Johri BM, Tiagi B. Floral morphology and seed formation in *Cuscuta reflexa*. Phytomorphology. 1952; 2: 162--180.
93. Truscott FH. Aspects of morphogenesis in *Cuscuta gronovii*. Am J Bot. 1966; 53: 739--750.
94. Lee KB, Park JB, Lee S. Morphology and anatomy of mature embryos and seedlings in parasitic angiosperm *Cuscuta japonica*. J Pl Biol. 2000; 43: 22--27.
95. García MA, Stefanović S, Weiner C, Olszewski M, Costea M. Cladogenesis and reticulation in *Cuscuta* sect. *Denticulatae* (Convolvulaceae). Org Divers Evol. 2018 18: 383--398.
96. Clayson C, García-Ruiz I, Costea M. Diversity, evolution, and function of stomata bearing structures in *Cuscuta* (dodders, Convolvulaceae): From extrafloral nectar secretion to transpiration in arid conditions. Perspect Plant Ecol Evol Syst. 2014; 16: 310--321.
97. Vogler DW, Kalisz S. Sex among the flowers: The distribution of plant mating systems. Evolution. 2001; 55: 202--204.
98. Goodwillie C, Kalisz S, Eckert CG. The evolutionary enigma of mixed mating systems in plants: Occurrence, theoretical explanations, and empirical evidence. Annu Rev Ecol Evol Syst. 2005; 36: 47--79.
99. Mulligan GA. Autogamy, allogamy, and pollination in some Canadian weeds. Can J Bot. 1972; 50: 1767--1771.
100. Primack RB. Reproductive effort in annual and perennial species of *Plantago* (Plantaginaceae). Am. Nat. 1979; 114: 51--62.
101. Graumann S, Gottsberger G. Reproductive strategies in allogamous and autogamous *Senecio* species. Lagascalia. 1988; 15: 673--679.
102. Guo Q, Brown JH, Valone TJ. Long-term dynamics of winter and summer annual communities in the Chihuahuan Desert. J Veg Sci. 2002; 13: 575--584.
103. Stewart FC, French GT. The perennation of the clover dodder, *Cuscuta epithymum* Murr. Torreya 1909; 9: 28--30.
104. Dean HL. Dodder overwintering as haustorial tissues within *Cuscuta*-induced galls. Proc Acad Iowa Sci. 1955. 61: 99--106.
105. Rao LN. Perennation in *Cuscuta reflexa* Roxb. New Phytol. 1938; 37: 474--477.
106. Meulebrouck K, Ameloot E, Brys R, Tanghe L, Verheyen K, Hermy M. Hidden in the host–Unexpected vegetative hibernation of the holoparasite *Cuscuta epithymum* (L.) L. and its implications for population persistence. Flora. 2009; 204: 306--315.
107. Thompson K, Grime JP. Seasonal variation in the seed banks of herbaceous species in ten contrasting habitats. J Ecol. 1979; 893--921.
108. Michaels HJ, Benner B, Hartgerink AP, Lee TD, Rice S et al. Seed size variation: Magnitude, distribution, and ecological correlates. Evol Ecol. 1988; 2: 157--166.
109. Guo Q, Brown JH, Valone TJ, Kachman SD. Constraints of seed size on plant distribution and abundance. Ecology. 2000; 81: 2149--2155.
110. Baker HG. Seed weight in relation to environmental conditions in California. Ecology. 1972; 53: 997--1010.
111. Venable DL, Brown JS. The selective interactions of dispersal, dormancy, and seed size as adaptations for reducing risk in variable environments. Am Nat. 1988; 131: 360--384.
112. Bekker RM, Bakker JP, Grandin U, Kalamees R, Milberg P, Poschlod P, et al. Seed size, shape and vertical distribution in the soil: indicators of seed longevity. Funct Ecol. 1998; 12: 834--42.
113. Maun MA. Adaptations enhancing survival and establishment of seedlings on coastal dune systems. Vegetatio. 1994; 111: 59--70.
114. Maestre FT, Cortina J, Bautista S, Bellot J, Vallejo R. Small-scale environmental heterogeneity and spatiotemporal dynamics of seedling establishment in a semiarid degraded ecosystem. Ecosystems. 2003; 67: 630--643.
115. Isselstein J, Tallowin JRB, Smith REN. Factors affecting seed germination and seedling establishment of fen‐meadow species. Restor Ecol. 2002; 10: 173--184.

**Supporting information captions**

**Table S1.** *Cuscuta* species previously studied for seed morphology and/or anatomy arranged alphabetically and indicating their publication source. “+” and “—” indicate presence or absence of data.

**Table S2.** Seed character dataset for *Cuscuta*. Refer to Table 1 for the character states. CP = Compression; S = Shape; ET = Embryo type; HP = Hilum position; HC = Hilum compression; Dep = Dry seed epidermis; Hep = Hydrated seed epidermis; ECS = Epidermal cell shape; OP = Presence of outer palisade layer; #C = number of embryo coils; L = Seed length (μm); W = Seed width (μm); ST = Seed thickness (μm); Hl = Hilum length (μm), HW = Hilum Width (μm); FL = Length of funicular scar (μm); ECD = Epidermal cell diameter (μm); EPT = Epidermal cell thickness (μm); Epidermal cell width (μm).

**Table S3.** Summary of basic statistics for quantitative characters of Cuscuta seeds.

**Table S4.** Pearson’s correlation summary for quantitative seed characters of Cuscuta.

**Table S5.** Seed size of three *Cuscuta* species studied and summary of statistical results.

**Figure S1.** Parsimony ancestral reconstruction of number of embryo coils in *Cuscuta* seeds. Embryos with more than 2.75 coils evolved multiple times in subg. *Grammica*.
