# Supplementary material for: Correction: Cuscuta seeds: Diversity and evolution, value for systematics/identification and exploration of allometric relationships
Source: PLoS One. 2020 Jul 1;15(7):e0235738. doi: 10.1371/journal.pone.0235738 (PMC7329106; doi:10.1371/journal.pone.0235738)
Supplement: S1 File — (DOCX) [file pone.0235738.s001.docx]

PLOS ONE, EDITORIAL OFFICE

22 May 2020

Dear Dr. Amico and Editorial Office,

We have been lucky to receive answers from 4 reviewers! The feedback provided by them has been helpful to improving this manuscript and we are grateful for their input. We have also responded in detail to all their comments made in the following pages. This rebuttal follows the format in which the points from the reviewers were left in the original order, in black, and our responses (in blue) were then inserted after each of those points.

**Reviewer #1**: Dear all,
The paper is interesting and should be publushed in Plos One. I have attached a file with some queries and suggestions, and I hope it helps to improve some parts of the paper.

Best wishes,
Alexander
Authors: ALL the suggestions made in the PDF uploaded by Alexander Sukhorukov have been incorporated in the new version of the manuscript (please see track changes version of manuscript).

**Reviewer #2**: It is "the most comprehensive study" on Cuscuta species, seeds and their associated classification and function.
Only suggestion is to add more information on following:
1, please define the "length", "width" and "thickness" in Table 1 line 12-14, it will increase the clarity. e.g., the first, second or third longest axis for length, width and thickness.

Authors: Okay; added a description for these characters in Table 1.

2, on the line of 189-190 to add seed year for those three species if the data available. Or seed year collected are comparable for the survival study since the longevity may play a role in their survival time.
Authors: Good catch! Also, now I noticed we had forgotten to add the herbarium vouchers for them. I included the information requested, plus the herbarium specimens used to Appendix S1.

**Reviewer #3:** This work is the most comprehensive study to date on the seed morphology and anatomy of the genus Cuscuta. Sampling includes about half of the species of the genus and all the sections currently recognized except for the small sect. Epistigma. A detailed revision of seed morphology and anatomy is complemented with the study of embryo morphology, number of seeds/capsule related to breeding systems, seed size and distribution range in N America, germination and seedling survival. All of them are analyzed in a robust phylogenetic framework. It is a robust work that will be a reference for future studies of seed biology in this economically and ecologically important genus of obligate parasitic plants. The paper is well written and the information presented clearly merits its publication in PlosOne. Only a few minor corrections might help to improve an already excellent paper in its current state.

- Lines 357-358; 394. There are four (not three) species described in this section, including C. psorothamnensis, sharing the embryo enlarged toward the radicular end typical of the section.

Authors: Reviewer’s observation is correct, but we did not include this 4^th^ species, *C. psorothamnensis*, in the anatomical study because it was described only in 2018. To mention it, we added a couple of sentences in the Discussions.

- It is not clear the anatomical structure in the hilar area. Most species have a continuous double palisade layer except for subgenus Monogynella and four (five?) species on Grammica in which the outer palisade layer is only present in the hilar area. Therefore the anatomy in subgenus Monogynella is two palysade layers and the epidermis above the endosperm in the hilar area.

Authors: No, ALL the species have 2 palisade layers in hilum area. The species indicated by the reviewers have 1 palisade layer only outside the hilum area. We know that this double/single palisade layers story is a bit confusing. We tried to clarify in the text and we have redone Fig 3, changing 3F with an image that clearly shows both palisade layers in the hilum area for *C. lupuliformis* (subg. *Monogynella*)

Figure 3E-F shows a stained thickened structure with the tracheid-like line between the external surface of the hilum and the endosperm. I could not find and explanation of what structure is that. Is it part of the endosperm or a swelling of the palisade layers? There is a significant increase in thickness of the inner palisade layer in the hilum pad but up two times (line 363). Therefore it cannot be the inner palisade layer because it is much thicker than that. This is confusing and should be explained more clearly. It would be useful to point with arrows the two palysade layers of the hilar pad.

Authors: We indicated now in the figure legend and in the text that is part of the water gap. The tracheid-like structures are embedded in a parenchyma which is not part of the endosperm. I hope that with the new figure and additional explanations, this is clearer now.

What is the line of the inner palysade layer (P1) shown in figure 1-O? Compare with the photograph of C. pacifica in Fig. 2. Comparing both images it could be interpreted that what is labeled as P2 in Fig. 1-O is actually the epidermis. In Fig. 1-N, what is the structure between the endosperm (En) and P1?
Authors: Excellent powers of observation! Fixed the labels in Fig. 1O.

-I might be wrong but I think there is a mistake in the legend of Fig. 5, lines 431-433. Taxa with more than 2.3 S/C were divided if they had "more of less" than 1.3 S/C. If the taxa had more than 2.3 S/C one of the groups cannot have less than 1.3 S/C. Add also a space in line 429 to separate 746 and 31 because it reads "746.31%".

Authors: Again, great catch -- we fixed this in the legend and improved the font size in Fig. 5.

Minor corrections:

-Sentence in lines 56-57. It is not clear what it means.

Authors: it seems nobody got what we were trying to say, so it was obviously an ambiguous phrasing. I rewrote that sentence.

-Correct sentence in lines 208-209.

Authors: okay.
-Line 471, add parenthesis to the list of references.

Authors: okay.

**Reviewer 4**

Authors: This review was quite laudatory and well-intentioned, but rather…unusual its recommendations. We apologize in advance for the profusion of text. Because the reviewer was quite exhaustive, our response also had to be similarly comprehensive. Thus, the rebuttal was divided in two parts: **1)** **the rebuttal of the letter** that the reviewer sent to the editor, which follows next, and **2)** **the rebuttal of the annotated manuscript** for which we answered the numerous comments directly in the document (attached separately; the file name is “response to reviewers 2). Several related comments from the annotated manuscript were discussed below.

Dear Editor,

The submitted manuscript seems to me a very important contribution to the knowledge of seed anatomy, morphology, and evolution and development in the genus *Cuscuta*, which is not only of great economic importance, but also very relevant for laying a foundation for further understanding the evolution and dispersal mechanisms of this parasitic plant, with impact on host-parasite interaction studies (ecology and evolution). It is a very comprehensive work, with a great quantity of data that must have required years of study, and analysed with appropriate methodological strategies, to the best of my knowledge. It will also be a good model for similar studies in the family Convolvulaceae, which are very much necessary.

Therefore, I am very supportive of the publication of these research results, for even with sampling limitations, it will leverage an array of other studies in the fields of plant systematics, ecology and evolution in this, and other, groups of plants.

Authors: thank you, we loved the two paragraphs above.

My most significant recommendations go towards the actual presentation of the results, i.e. how the manuscript is written and structured. The importance of making these data and analyses available is undeniable, but I would strongly recommend to present it in a more effective way.

I have felt that the research hypothesis and how the applied methodology will address them are not laid out clearly enough, and in my view the contents are mixed up both in the Introduction, Methodology and Discussion. A more structured organization of the contents would allow summarizing some parts thar are overlapping between sections, and expanding on some discussions which would be interesting to extend.

As I understand it, this study is decomposed in the following components:

1) What is the variation? Documenting the variation in seed morphology and anatomy and coding this variation for further analysis; also, explicitly laying out the taxonomic and biogeographic scope of the study (if it is not the same for each section, I do not see a problem, but it needs to be explicitly defined from the outset).
2) How has this variation evolved in the group? Testing character evolutionary hypothesis in a molecular phylogenetic framework
3) Can this variation contribute to the systematics of the group? Given the character evolution, can synapomorphies be found for particular clades, and are they useful or not for diagnosing taxa of any particular rank?
4) Character correlations – do certain traits correlate to each other, and possibly why?
5) Additional information about seed development

Authors: Based on the above, it is possible that the reviewer misunderstood or placed a different weight on some of the objectives clearly outlined at the end of the introduction. The findings of this paper can be divided into two main parts:

A) Diversity and evolution of seed traits (both morphological and anatomical).
B) An attempt to connect findings from (A) with a functional explanation (“form and function”).

First, I notice that that all the “components” understood by the reviewer (1-5) belong to our part A; the functional aspect was not perceived. Second, we understand variation as having multiple dimensions not only epistemologically but also pragmatically: there is a descriptive part -- of the morphological, anatomical and developmental features found, and then there is an analytical part in which data were analyzed both in terms of their statistical meaningfulness (in this particular case correlations between traits) and evolutionary history (reconstructing ancestral character states). There was no apriori evolutionary hypothesis (or hypotheses) in this respect for part A (see 2). Instead, the study generated such hypotheses based on the character evolution, which then it tried to connect both in the results and the discussions with the functional, allometric aspects in the second part (B). “Can this variation contribute to the systematics of the group?” This can only result from all the layers of diversity surveyed and analyzed (part of the different Results sub-sections), which was then clearly discussed in a separate section of “Discussions”. 5) Additional information about seed development. This is not something “additional”; seed anatomy and development are just as important as morphology; we may see perceive them as “different” because of the different methodologies we use to reveal them, but in fact, they are aspects of a unified form architecture of the seeds.

The clear structure of contents would allow greater transparency and clarity in the communication of the hypotheses, and interpretation of the results. For instance, I understand that the characters explored did not contribute in a meaningful way to the systematics of the group. However, this is a result in itself, and further corroborates the already documented homoplasy present in so many characters in family Convolvulaceae. This needs to be openly addressed and discussed, as the other research questions.

Authors: Yes, and by explicitly tracing character evolution of both morphological and anatomical traits, including by revealing their convergent evolution and extensive homoplasy in results, we did exactly that. I fail to see how this was not been clearly addressed in “Results” and then thoroughly approached in the “Discussions” (in its own subsection, no less)

My suggestion for a re-structuring of the manuscript is to

- offer the objectives in the form of research questions or scientific problems (in the way they are presented, they seem more like a narrative of the methodology)

Authors: Objectives are not supposed to be “philosophical” (e.g., questions); they are supposed to clearly delineate the research done. “To do” is a verb; it implies an action. In the case of research that also assumes “how things are done”, which may allude to aspects of methodology.

- Present the results and discussion as answers to these research questions, therefore following a particular order of contents within each section, e.g. documentation of the variation, evolutionary analyses, value of characters for systematics and correlations between characters

Authors: It took a long time to understand what the reviewer meant by “the research questions or scientific questions” because it was impossible from the comments above and others. This was possible only after I found an example in the suggestions made within the manuscript -- please see below the answer to comment #10.

For further details, I have added comments throughout the manuscript draft, but several of those will relate to this general organization of contents that I have laid out, and which I think hinders the value of this publication as is.

In summary, I recommend the manuscript to be accepted with major revisions, and I would be happy to revise an improved version.

**----------------------------------------------------------------------------------------------------------------**

**Some of the most important comments that reviewer #4** made (in black) in the annotated manuscript (attached separately).

[Comment #5 in the Introduction] I would suggest that the Introduction is clearly organized in specific research questions, laying out previous literature on the subject, your hypothesis, and how you went about to test those hypothesis. I think the Introduction becomes unclear and ambiguous, when your research questions are not laid out explicitly and addressed one by one.

Authors: This not a student thesis in which the introduction can be subdivided into subsections addressing particular topics, gradually reaching to research question/hypotheses. This is an article -- a complex one -- and the intro had to rapidly identify gaps of knowledge in such a way objectives could be formulated in a relatively brief text. “Decomposing” this article into narrow research questions, as mentioned by the reviewer in the letter above and below, would entail a couple of dozen queries from both parts A & B (please see above), which is impractical.

[Comment # 7 in the introduction] Since one of the aspects of the work is the relevance of seed characters for systematics of the genus, I think it is essential to give an overview of the infrageneric classification of the genus, and highlight issues, if there are any. What characters are used for infrageneric classification and why would seeds be investigated to help at this level?

Authors: These are generalities published in at least 10 papers; they are not directly connected to our objectives revolving around seeds. We mentioned the scarcity of characters available for systematics of *Cuscuta* (in general) in the introduction and discussed the relevance of seeds to systematics of the genus in a distinct section called: “*Potential use of seed characters for systematics and identification of Cuscuta species”*. Again, this is not a thesis, and it cannot afford the luxury on tangents within the introduction because that would obscure the tracks that we tried to trace to rich our objectives.

[Comment # 10. In the introduction] Ok, so these are separate research questions that, I think, are not clearly laid out.
1) alpha-taxonomy: Are seed characters taxonomically informative at species level?
2)character evolution: How have these characters evolved within the genus?

3) systematics: Can synapomorphies be identified at higher taxonomic levels (e.g. subgenera or sections), which could be informative for infra-generic systematics?

Authors: Here are some examples of “research questions”. The reductionist approach of defining narrow questions is not practical here because: 1) there would be too many of them (the above are just a few possibilities as there are also similarly valid morphological, anatomical, developmental, functional “questions” that one could ask), and 2) multiple such “questions” can be solved by common objectives because their interrelating nature.

[Comment # 24. In Material and methods] I would definitely separate character evolution from any statistical correlations calculated between traits, as they address different research questions. I think mixing them up in a single section is somewhat confusing/ambiguous.

Authors: The correlation between characters is deeply connected with the character evolution because characters may evolve together. For example, length/width/thickness, although treated here as different seed characters, are in fact different metrics that express what we could call “seed size”. As seeds evolve, these traits are strongly correlated because they probably evolve together (or vice-versa!). The biological source of the correlation is complex: genetic, regulatory, allometric, etc., but the bottom line is that selection acts on the entire seed, as an organ part of an organism, etc., and our distinction of “characters” is just for the purpose of analyses. Mesquite has a module to test correlations within the phylogeny only for binary characters; for the quantitative traits it was easier to use regular statistical correlations.

**Closing thoughts for reviewer #4**

While some of the detailed comments made in the annotated manuscript were useful (please the attached document, “response to reviewers 2”), I found that the constant attempt to intellectually redefine this paper based on an unilateral understanding of what this study is (or should be) about, was not constructive. This is not a taxonomic paper; systematics usefulness is just a part of it, which was subordinated to a larger context: the evolution of form and function in *Cuscuta* seeds.

The role of the article reviewer, in my humble opinion, is to safeguard the validity of approaches used and to improve a paper in its existing form. Adding, redoing, removing, restructuring, rewriting should be suggested to improve, not to divert or substitute the though process of the author. Ultimately, the reviewer is anonymous and it is the author who bears the responsibility for a published paper. Even to a collaborator or co-author, in a similar situation, I would ask them to take the leadership of the writing process (if they think they can do a better job), and if after the due discussions or polemics, the opinions remain irreconcilable from an intellectual point of view, I would regretfully decline the collaboration or co-authorship.

Sincerely,

Mihai Costea

Department of Biology;

Wilfrid Laurier University;

75 University Avenue West,

Waterloo, ON, N2L 3C5, Canada

Phone: 519 884-0710; 3407 (office) or 2887 (lab)
